# GABAergic CA1 neurons are more stable following context changes than glutamatergic cells

**DOI:** 10.1038/s41598-022-13799-6

**Published:** 2022-06-20

**Authors:** Peter J. Schuette, Juliane M. Ikebara, Sandra Maesta-Pereira, Anita Torossian, Ekayana Sethi, Alexandre H. Kihara, Jonathan C. Kao, Fernando M. C. V. Reis, Avishek Adhikari

**Affiliations:** 1 Department of Psychology, University of California, Los Angeles, Los Angeles, CA 90095 USA; 2 Centro de Matemática, Computação e Cognição, Universidade Federal do ABC, São Bernardo do Campo, SP 09606-070 Brazil; 3 Department of Electrical and Computer Engineering, University of California, Los Angeles, Los Angeles, CA 90095 USA

**Keywords:** Hippocampus, Spatial memory, Learning and memory

## Abstract

The CA1 region of the hippocampus contains both glutamatergic pyramidal cells and GABAergic interneurons. Numerous reports have characterized glutamatergic CAMK2A cell activity, showing how these cells respond to environmental changes such as local cue rotation and context re-sizing. Additionally, the long-term stability of spatial encoding and turnover of these cells across days is also well-characterized. In contrast, these classic hippocampal experiments have never been conducted with CA1 GABAergic cells. Here, we use chronic calcium imaging of male and female mice to compare the neural activity of VGAT and CAMK2A cells during exploration of unaltered environments and also during exposure to contexts before and after rotating and changing the length of the context across multiple recording days. Intriguingly, compared to CAMK2A cells, VGAT cells showed decreased remapping induced by environmental changes, such as context rotations and contextual length resizing. However, GABAergic neurons were also less likely than glutamatergic neurons to remain active and exhibit consistent place coding across recording days. Interestingly, despite showing significant spatial remapping across days, GABAergic cells had stable speed encoding between days. Thus, compared to glutamatergic cells, spatial encoding of GABAergic cells is more stable during within-session environmental perturbations, but is less stable across days. These insights may be crucial in accurately modeling the features and constraints of hippocampal dynamics in spatial coding.

## Introduction

Hippocampal CA1 pyramidal glutamatergic cells strongly represent an animal’s allocentric location. Neurons that encode spatial information are called ‘place cells,’ and these cells become more active when an animal enters a specific area of an environment called a ‘place field’^1^.

Interestingly, CA1 pyramidal place cells remap in predictable ways following contextual changes. For example, lengthening or shortening a rectangular enclosure may accordingly stretch or compress the place field representation of that environment^2–4^. Similarly, changing the location of proximal cues or rotating the environment can also rotate pyramidal spatial maps, showing that a large fraction of CA1 principal cells is dominated by local spatial frames^5–15^. The advent of calcium imaging revealed that remapping also occurred in chronically recorded CA1 principal cells, which exhibited significant turnover of active cells across days^16^, despite also maintaining a degree of stability of spatial representation for several days^6,17–20^.

Recent work has shown that CA1 interneurons also represent location within an environment^21–25^. Despite these pioneering reports, classic hippocampal experiments, such as rotation of local cues or re-scaling of the environment have not been done while recording interneuron activity. Furthermore, the dynamic changes of interneuron spatial encoding across days are also not well-characterized. Consequently, it is not known if these cells have a rigid or flexible spatial code within sessions or across days. The extent and nature of the influence of local cues and geometry on CA1 interneuron activity is also not known.

While interneuron and glutamatergic cell types have been shown to represent a range of behavioral variables including position^1^ and running speed^26–34^, few studies have conducted an in-depth comparison of their various encoding properties, and none, to our knowledge, have contrasted these properties across spatial manipulations or multiple recording days—an analysis afforded only recently by modern calcium imaging techniques. For instance, do CA1 interneurons remap in a predictable manner, similar to pyramidal cells in this region, following environmental distortions? Can speed be predicted on a moment-to-moment basis by both cell types? Are CA1 interneurons consistently active across days, and do they maintain consistent place coding? Furthermore, to our knowledge, the stability of interneuronal spatial encoding has not been directly compared to that of pyramidal cells, and, on a more fundamental level, it is unclear if these cells maintain consistent activity across days or after alterations of local cues.

To answer these pressing questions, we obtained chronic calcium recordings of CA1 glutamatergic and GABAergic ensembles, which allowed us to compare their cell-type specific activity during naturalistic exploration as well as across various spatial manipulations and multiple recording days. While the majority of previous interneuronal studies have utilized electrophysiological recording, there is a vast and growing body of research that has targeted interneurons with more recently-developed calcium imaging techniques^25,35–41^; despite the comparatively high baseline firing rate of interneurons to that of pyramidal cells, the resulting data have been used to conduct temporally precise analyses, such as the delineation of ensemble response properties and encoding during in vivo exploration and goal-directed learning tasks^25,36–40^. These numerous prior reports demonstrate the feasibility and advantages of using calcium imaging to study interneuron activity. Thus, using this method, our data show that, compared to glutamatergic neurons, CA1 GABAergic cells show complex differences in spatial encoding: GABAergic place cells have higher spatial information, and they exhibit less remapping induced by contextual changes. However, GABAergic cells also paradoxically show increased remapping and cell turnover across days. Intriguingly, while spatial encoding in GABAergic cells is unstable across days, encoding of speed remains consistent during this time period. These results provide important insights and constraints on models of hippocampal function.

## Results

**Figure 1:**
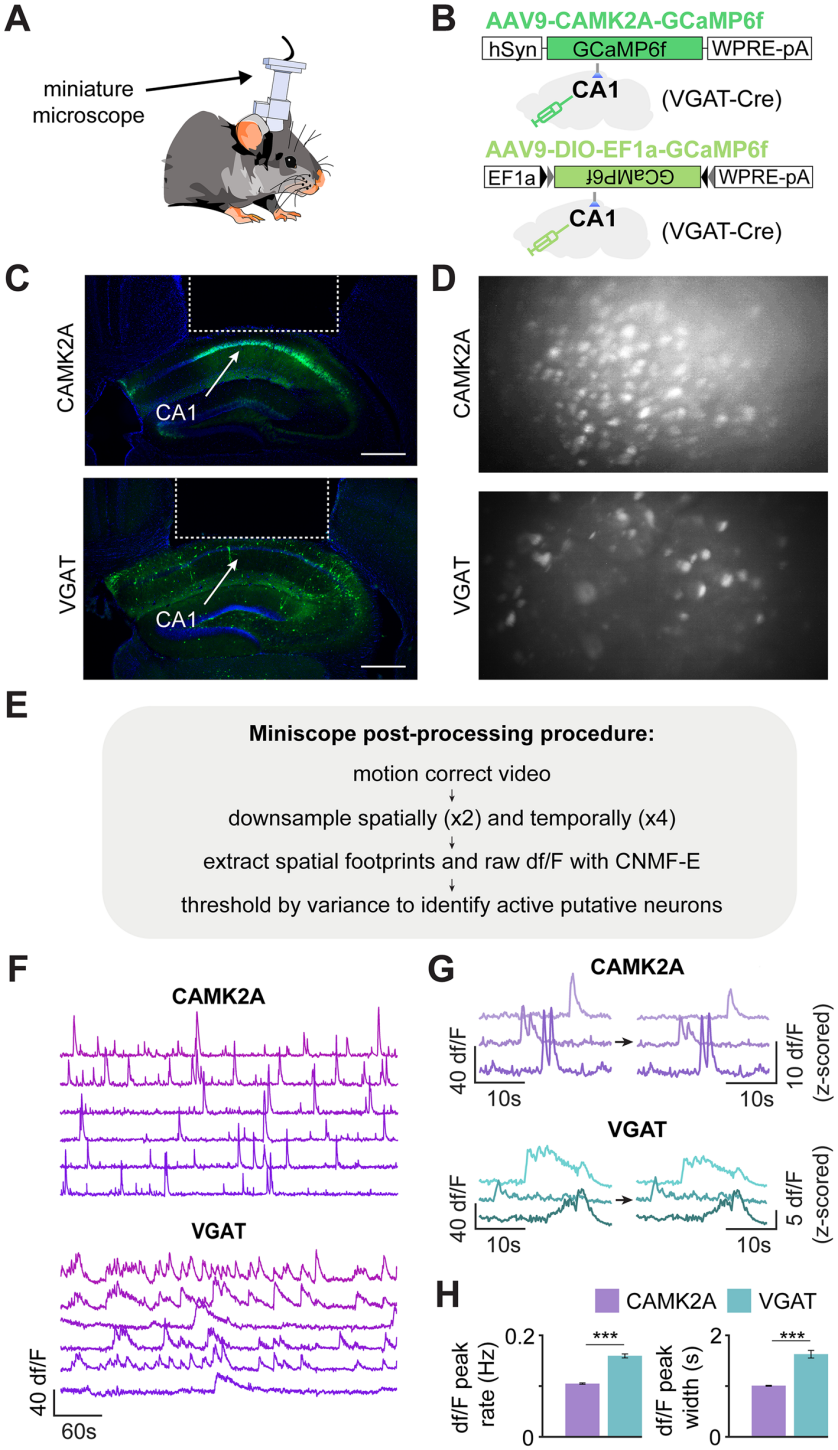
Recording calcium transients in CAMK2A- and VGAT-expressing cells in dorsal hippocampal area CA1. (**A**) Scheme showing mouse with miniaturized microscope to record calcium transients from either CAMK2A- or VGAT-expressing cells in dorsal hippocampal area CA1. (**B)** Scheme showing viral constructs used to target expression of the calcium indicator GCaMP6f to either CAMK2A- or VGAT-expressing cells in CA1. VGAT-cre driver mice were used in both cases. (**C**) Representative histology showing expression of GCaMP6f in CAMK2A- and VGAT-expressing cells. The location occupied by the miniaturized microscope lens is outlined by a dashed white line (scale bar = 500 μm). (**D**) Representative maximum projection showing views of CAMK2A- (top) and VGAT- (bottom) expressing cells through the implanted lens. (**E**) The miniscope post-processing procedure. (**F**) Representative traces of CAMK2A (top) and VGAT (bottom) cells from area CA1 of the dorsal hippocampus. (**G**) Shown are example CAMK2A and VGAT traces before and after z-scoring. (**H**) Bars represent the rate of calcium peaks (left; CAMK2A rate = 0.11 ± 0.001 peaks/s; VGAT rate = 0.17 ± 0.004 peaks/s) and peak width (right; CAMK2A peak width = 0.77 ± 0.007 s; VGAT peak width = 1.34 ± 0.09 s) for CAMK2A and VGAT cells. (two-sample t-test; CAMK2A n = 2303 (from 8 mice); VGAT n = 937 (from 12 mice); (left) t-statistic = − 17.7, (right) t-statistic = − 10.1). ***p < 0.001.

**Figure 2:**
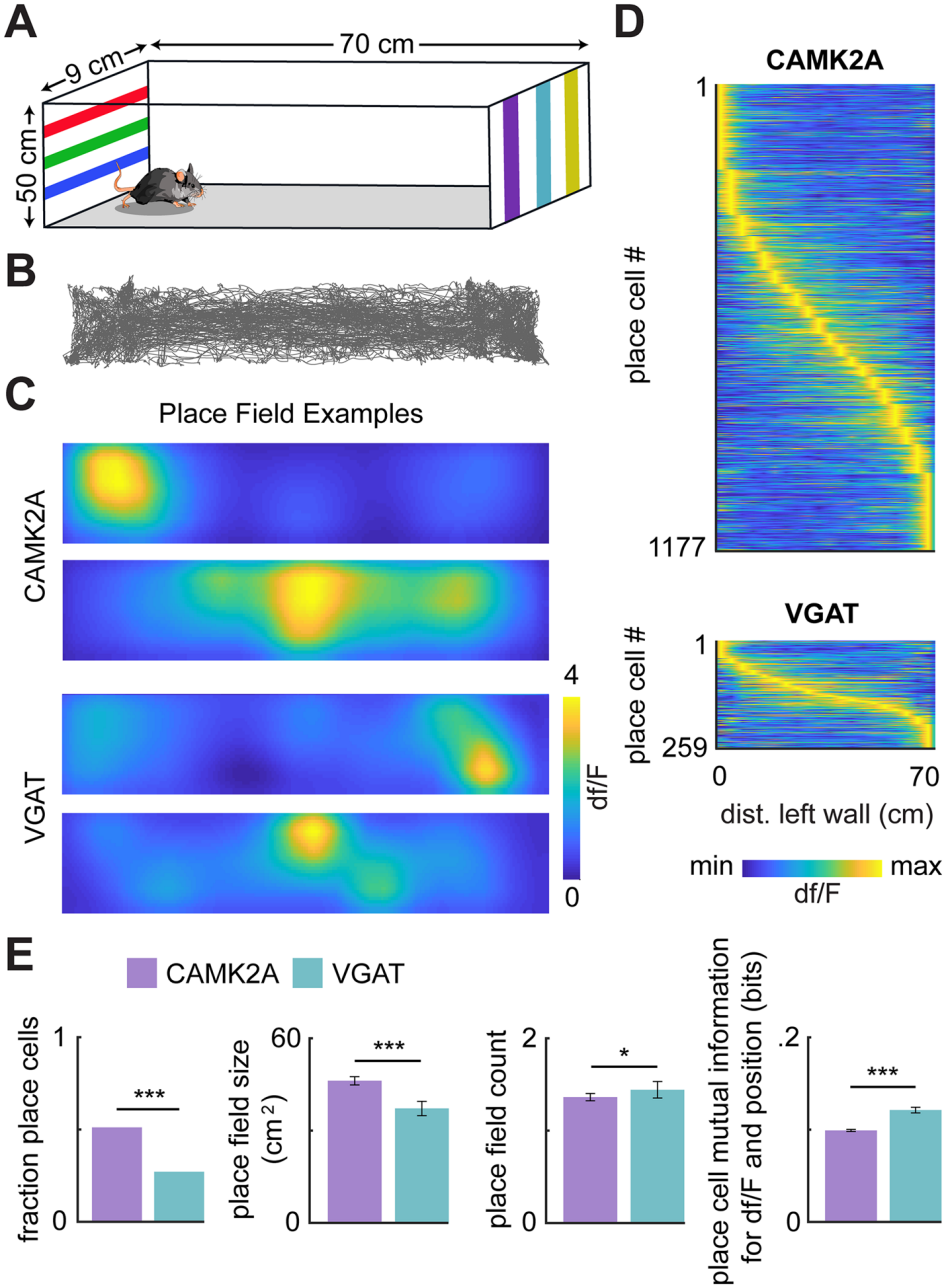
Characterization of firing properties of CAMK2A- and VGAT-expressing CA1 cells. (**A**) Scheme of environment used for recordings. (**B**) Example path for a single session. (**C**) Heatmaps showing firing rate maps of example place cells. Higher activity is indicated by warmer colors. (**D)** Colormap showing neural activity of all recorded place cells for CAMK2A (top) and VGAT cells (bottom). Cells are sorted by the location of their place field (CAMK2A n = 1177, VGAT n = 259). (**E**) Quantification of spatial representation metrics for CAMK2A (purple) and VGAT (turquoise) cells. Compared to VGAT cells, a higher fraction of CAMK2A cells are classified as place cells (fraction of putative neurons categorized as place cells: CAMK2A = 0.51, VGAT = 0.27). Among cells classified as place cells, VGAT cells show a smaller place field size (place field size: CAMK2A = 46.21 ± 1.35 cm^2^, VGAT = 37.24 ± 2.31 cm^2^) a slightly greater place field count (place field count: CAMK2A = 1.36 ± 0.04, VGAT = 1.43 ± 0.09) and higher mutual information (bits: CAMK2A = 0.10 ± 0.001, VGAT = 0.12 ± 0.003) than CAMK2A cells. (statistics and sample sizes: (left) Fisher’s exact test, CAMK2A: place cell n = 1177, non-place cell n = 1126 (from 8 mice); VGAT: place cell n = 259, non-place cell n = 697 (from 12 mice); (middle/left) Wilcoxon rank-sum test; CAMK2A n = 1177 (from 8 mice), VGAT n = 259 (from 12 mice), z = 6.38; (middle/right) Wilcoxon rank sum test; CAMK2A n = 1177 (from 8 mice), VGAT n = 259 (from 12 mice), z = − 2.34; (right) Wilcoxon rank sum test; CAMK2A n = 1177 (from 8 mice), VGAT n = 259 (from 12 mice), z = − 6.44).***p < 0.001, *p < 0.05.

To compare the spatial representations of Calcium/Calmodulin Dependent Protein Kinase II Alpha (CAMK2A)- and vesicular GABA transporter (VGAT)-expressing cells in the dorsal hippocampal area CA1, we used miniature microscopes^42^ to record the calcium activity from the pyramidal layer of two cohorts of VGAT-cre mice with GCaMP6f. localized to each cell type (Fig. 1A–G). VGAT-expressing cells displayed increased baseline levels of neural activity compared to principal cells expressing CAMK2A, as reported previously (Fig. 1H)^21–24,43^. For recordings from VGAT cells, these mice were injected with AAV9-DIO-GCaMP6f virus, while for recordings from CAMK2A glutamatergic cells, VGAT-cre littermate mice were injected with AAV9-CAMK2A-GCaMP6f. We then performed an experiment in which mice were allowed to freely explore a spacious enclosure (Fig. 2A; 70 × 9 × 50 cm), allowing ample room to quantify the hippocampal spatial representations of each cell type (Fig. 2B–D; see “Linear track” section of “Methods” for more details). Thus, the experiments had no explicit trial structure or task, as they consisted of free, naturalistic exploration. Mice thoroughly explored the enclosure (see representative behavioral track in Fig. 2B). Putative neurons were extracted as reported previously^44^. A total of 2303 CAMK2A and 937 VGAT neurons were recorded in a single session for mice exploring a linear track (CAMK2A sessions n = 8, VGAT sessions n = 12). Among these cells, 1177 CAMK2A and 259 VGAT cells were categorized as place cells (Fig. 2C,D; Supplemental Table 1). Thus, a significantly greater fraction of CAMK2A than VGAT neurons was categorized as place cells (fraction of putative neurons categorized as place cells: CAMK2A = 0.51, VGAT = 0.27; Fig. 2E; see “Methods” for ‘place cell’ definition). Additionally, compared to CAMK2A cells, VGAT place cells exhibited a slightly smaller place field size and greater mutual information between spatial location and calcium activity (Fig. 2E) indicating that, while there are fewer place-encoding VGAT cells, this subset may encode spatial location to a more specific degree than CAMK2A place cells.

**Figure 3:**
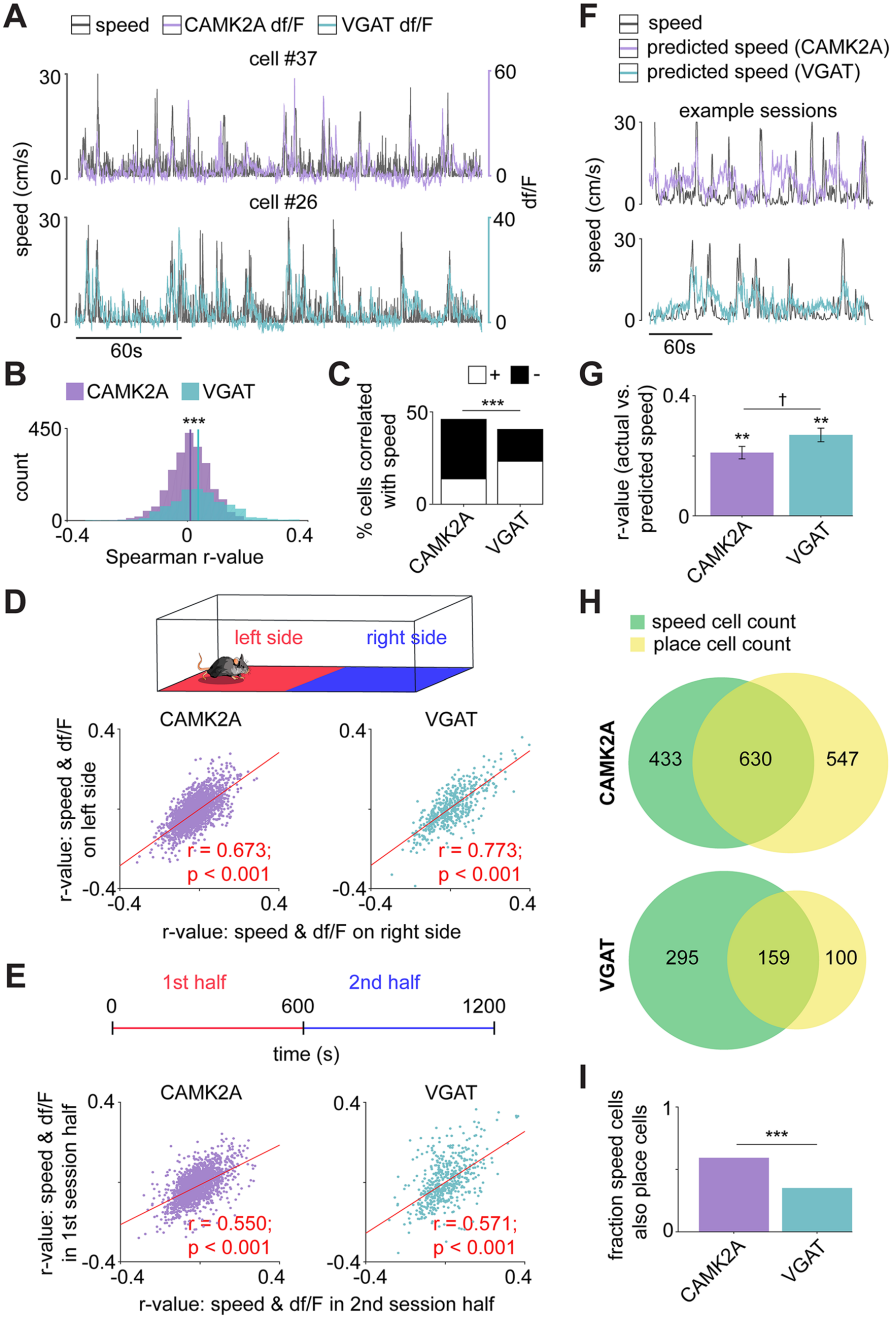
Decoding speed with neural activity. (**A**) Example representative CAMK2A (purple, top) and VGAT (turquoise, bottom) df/F traces of cells that correlated with speed. (**B**) Histograms show the CAMK2A and VGAT distributions of Spearman r-values for df/F and speed for all cells (mean r-values: CAMK2A = 0.003, VGAT = 0.030). (statistics and sample sizes: Wilcoxon rank sum test, CAMK2A cell n = 2303 (from 8 mice); VGAT cell n = 937 (from 12 mice), z = − 9.66) (**C**) Quantification of cells whose activity is positively and negatively correlated with speed [statistics and sample sizes: Fisher’s exact test; CAMK2A + cell n = 317 (13.8%), CAMK2A − cell n = 746 (32.4%); VGAT + cell n = 317 (33.8%), VGAT − cell n = 137 (14.6%)]. (**D**) (top) Diagram of the enclosure split into equally-sized left and right sides. (bottom) The correlation of speed and df/F was calculated separately for samples recorded in the left and right sides of the enclosure for all recorded CAMK2A and VGAT cells. The resulting r-value vectors were then correlated for matched cells on the left and right sides of the enclosure. (Spearman correlation; CAMK2A n = 2303; VGAT n = 956) (**E**) (top) Diagram showing the 20-min session was divided into first and second halves. (bottom) Similar to (**D**) but instead correlating speed and df/F across session session halves rather than enclosure sides. (Spearman correlation; n same as (**D**, **F**) A generalized linear model (GLM) was implemented for each mouse, with neural activity as the predictor variable and speed as the response variable. Traces show examples of observed and GLM-predicted speed. (**G**) Bars show the mean Spearman correlation for the observed and GLM-predicted speed. While the actual and predicted speed shows a high correlation for both cell types, VGAT cells were better predictors of this behavioral measure (statistics and sample sizes: Wilcoxon sign rank and rank-sum tests; CAMK2A sessions n = 8, mean r-value = 0.21 ± 0.02; VGAT sessions n = 10, mean r-value = 0.27 ± 0.02, see “Methods” section entitled “Speed Decoding” for mouse exclusion criteria). (**H**) Venn diagrams depict the overlap of CAMK2A and VGAT cells that were categorized as speed cells (green) and place cells (yellow). (**I**) Bars show the fraction of speed cells that were also categorized as place cells for CAMK2A (0.59) and VGAT (0.35) cohorts. A greater proportion of CAMK2A speed cells encoded both features. (statistics and sample sizes: Fisher’s exact test; CAMK2A: speed cell only n = 433, speed and place cell n = 630 (from 8 mice); VGAT: speed cell only n = 295, speed and place cell n = 159 (from 12 mice)) ***p < 0.001, **p < 0.01, ^†^p = 0.079.

In order to determine if these CAMK2A and VGAT-expressing neurons encode other behavioral metrics, we classified individual neurons as positively- or negatively-correlated with speed. To separate encoding of speed from the encoding of place, we first regressed out neural activity correlated with position (see “Methods”). We compared the Spearman correlation of each cell’s activity with mouse speed, then compared this actual r-value with a distribution of r-values computed from circularly shuffled data (see “Methods”). Cells with Spearman absolute r-values greater than 95% of the resulting shuffled distribution were considered to be significantly correlated with speed. A substantial portion of each cell type showed significant correlation with speed (Fig. 3A–C). As is apparent in Fig. 3C, the proportions of positively and negatively correlated cells differed between cell types; more CAMK2A cells were negatively correlated with speed, while more VGAT cells were positively correlated with speed.

While we had initially regressed the contribution of position from each cell’s activity prior to speed cell categorization (see “Methods”), it was also crucial to further verify that this speed encoding (1) did not depend on location within the enclosure and (2) was consistent across the session. To do so, we first calculated the correlation of speed and df/F separately for (1) left and right sides of the enclosure and (2) first and second session halves (i.e., the initial and latter 10-min epochs of a 20-min session). We then correlated these r-values across enclosure sides and session halves; there was a strong correlation for each cell type and pairing (Fig. 3D–E), indicating this speed encoding persisted across both the length of the enclosure and the duration of the 20-min recording session. The finding that speed correlations persisted after regressing out correlations with position, and that speed encoding was correlated across left and right halves of the environment indicate that both VGAT and CAMK2A cells encode speed in a manner that is not directly caused or confounded by the encoding of place.

To determine the comparative strength of speed encoding per mouse, we implemented a generalized linear model (GLM) to predict speed from the raw neural data, from which we had regressed out neural activity correlated with position. To account for the difference in number of putative neurons per mouse, we first found the mouse with the smallest number of recorded neurons across all mice and randomly selected that number of neurons to use for this decoding analysis (The mouse with the fewest number of recorded neurons had neuron count *n* = 32; thus, all mice had 32 or more neurons recorded, see “Methods” for more details). This analysis was performed 100 times, selecting a different neuronal subset of 32 neurons for each iteration. The mean Spearman correlation of the actual and GLM-predicted metric (testing data only) was significantly greater than the null distribution built from circularly shuffled speed across each tested mouse and in both cohorts. Thus, speed was significantly encoded by both CAMK2A and VGAT cell populations for all mice. However, the strength of this encoding differed by cell type, and speed was better predicted by VGAT neural data compared to CAMK2A cells (Fig. 3F,G). While there was a substantial overlap of cells that encode both speed and place for CAMK2A and VGAT cohorts (Fig. 3H), a significantly greater proportion of CAMK2A than VGAT speed cells were also categorized as place cells (CAMK2A = 0.59, VGAT = 0.35; Fig. 3I). Thus, VGAT cells show somewhat greater specialization than CAMK2A cells in coding these two behavioral variables.

Qualitative observations of the calcium activity for each cell type suggested that some degree of ensemble coactivity, or synchrony, occurred during spatial navigation. Such synchrony has previously been reported for hippocampal CAMK2A neurons during sharp wave ripples, characterized as 100–250 Hz oscillations within short 50- to 400-ms periods that usually occur during low speed epochs^45–51^. To compare the behavioral correlates of this phenomenon across cell types, we first classified this coactivity, termed ‘population events,’ as samples for which the number of coactivated neurons exceeded the mean by at least 4 SDs (see “Methods”; Supplemental Fig. 1A,B). Using this definition, we found an average of 178 ± 6.06 and 141.42 ± 18.15 population events for CAMK2A and VGAT cells across a 20-min session, indicating they occur frequently enough to warrant further investigation. Overlaying these population event times on a plot of instantaneous mouse speed, it became apparent that this synchrony arose at distinct speed ranges; CAMK2A and VGAT population events were observed during, respectively, low and high-speed epochs (Supplemental Fig. 1A,B). Indeed, when comparing the cumulative percent of population events to occur at increasing speed ranges, significantly more CAMK2A events were classified at lower speeds than VGAT events (Supplemental Fig. 1C). Furthermore, the mean speed during population events was significantly lower for CAMK2A than VGAT cells (Supplemental Fig. 1D), indicating this coactivity is occurring at distinct times for CAMK2A and VGAT cells. A large percentage of both CAMK2A and VGAT cells participated in these events (Supplemental Fig. 1E); however, participation did not correspond with any discernible place encoding dynamics. For instance, there was no difference in either place field width or mutual information for population event active and non-active cells across either cell type (Supplemental Fig. 1F).

**Figure 4:**
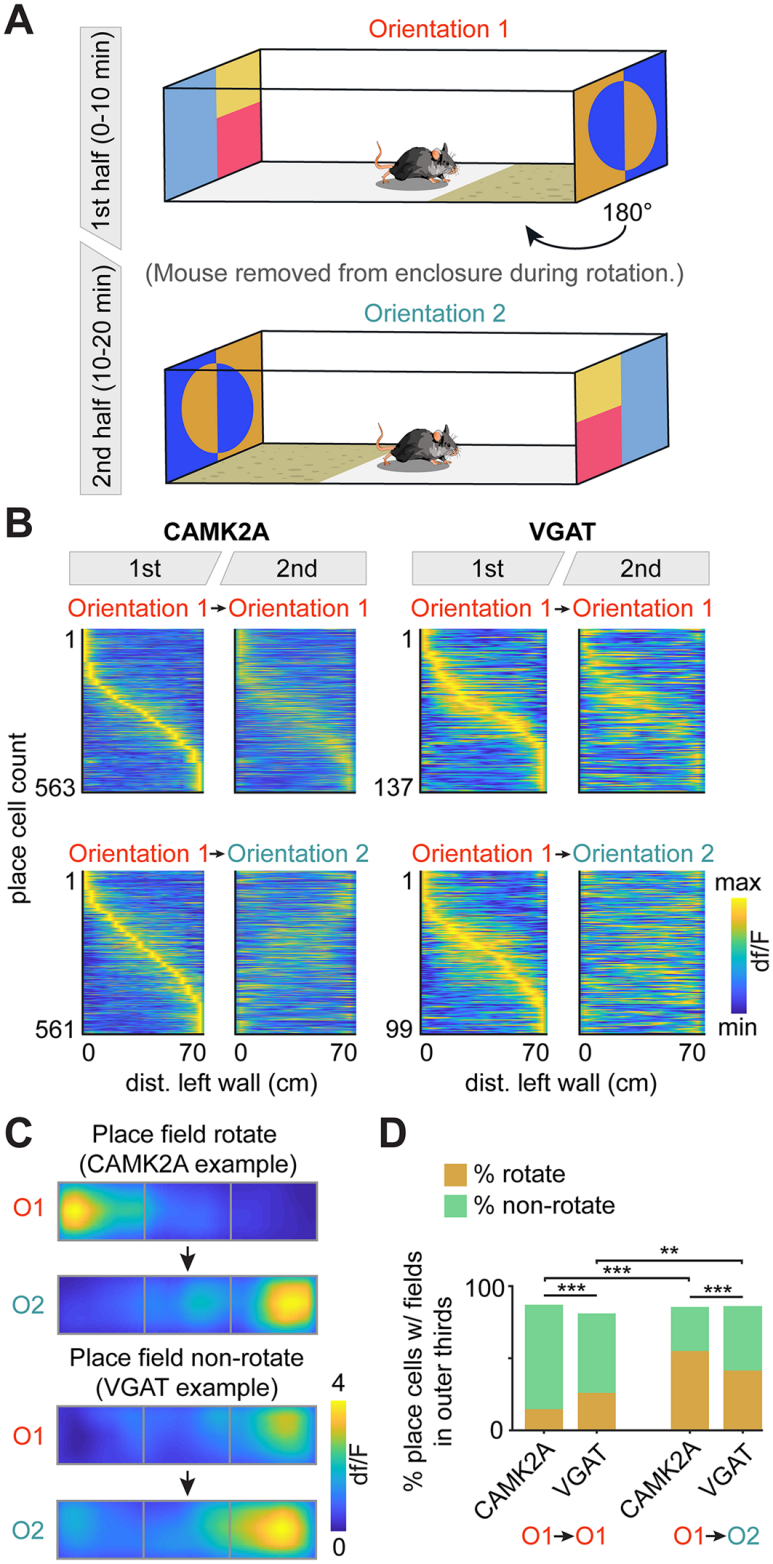
CAMK2A cells are more likely than VGAT cells to rotate their place fields following an environmental rotation. (**A**) Recordings were obtained in a box before and after rotating the box by 180 degrees (these recordings correspond to the orientation 1-to-2 sessions). The mouse was removed during the rotation. Distal visual landmarks outside the box were not rotated. In control sessions on separate days, the box was not rotated (these correspond to orientation 1-to-1 sessions). (**B**) Heat maps showing firing patterns of cells in sessions without (top, orientation 1-to-1) or with rotation (bottom, orientation 1-to-2). Cells are aligned in all plots relative to their firing location in orientation 1. Notice that in the orientation 1-to-1 session (top), both CAMK2A and VGAT cells tend to maintain their position of firing. However, in orientation 1-to-2 rotated sessions (bottom), CAMK2A cells tended to rotate their place fields (i.e., cells that were active at 0 cm in orientation 1 tended to be active at 70 cm in orientation 2) while VGAT cells did not rotate consistently. A cell was included in this analysis if it was categorized as a place cell in the first session half. [CAMK2A orientation 1-to-1 n = 563 (from 8 mice), VGAT orientation 1-to-1 n = 137 (from 12 mice), CAMK2A orientation 1-to-2 n = 561 (from 8 mice), VGAT orientation 1-to-2 n = 99 (from 11 mice)] (**C**) Shown are examples of CAMK2A and VGAT cell place fields for the orientation 1-to-2 rotated session. The top example cell rotates its place field from the leftmost third to the rightmost third of the enclosure (thirds designated by gray boxes) while the bottom cell maintains its place field position, irrespective of rotation. (**D**) Bars depict the percentage of place cells with place fields in the outer thirds of the enclosure that either rotated their place field to the opposite outer third (as in **C**, top) or maintained their place field position, irrespective of rotation (as in **C**, bottom). Place cells with fields in the middle third were excluded from this analysis, as it was not possible to ascertain if place fields in the middle quadrants rotated with the environment. A greater proportion of CAMK2A cells maintained their field location for orientation 1-to-1 sessions than VGAT cells, while a greater proportion of CAMK2A cells rotated their field location for orientation 1-to-2 sessions than VGAT cells. Rotation of the context significantly increased the proportion of both CAMK2A and VGAT cells that rotated their place fields [Statistics and sample sizes: Fisher’s exact test, Orientation 1-to-1: CAMK2A rotate n = 83 (14.6%), non-rotate n = 411 (72.5%; from 8 mice), VGAT rotate n = 43 (26.1%), non-rotate n = 91 (55.2%; from 12 mice); Orientation 1-to-2: CAMK2A rotate n = 261 (55.1%), non-rotate n = 145 (30.6%; from 8 mice), VGAT rotate n = 67 (41.1%), non-rotate n = 72 (44.2%; from 11 mice)]. ***p < 0.001, **p < 0.01.

Having verified that both CAMK2A and VGAT cells encode spatial location to a significant degree (Fig. 2), we sought to determine whether VGAT cells respond to well-studied environmental perturbations in a manner similar to that previously reported for principal neurons^5–15^. Prior data from a body of classic place cell research have shown that many CA1 principal cells rotated their place fields when the experimental enclosure was rotated. A recent work^25^ also demonstrated that, similar to CAMK2A cells, VGAT cells tend to remap in completely novel contexts. We thus compared how VGAT and CAMK2A cells responded to rotations of the environment. To do so, we performed a second experiment; mice were habituated to a new enclosure marked with different colored tape patterns on the left and right extremities. This coloring pattern provided prominent proximal cues that were distinct on each side of the box. Mice freely explored the box over a period of 3 days (70 × 15 × 50 cm; see “Rotated linear track” in “Methods”), again with no explicit trial structure or task. On the next two daily sessions, the box was rotated either 0 or 180 degrees, halfway through the session (Fig. 4A). A total of 2213 CAMK2A and 874 VGAT neurons were recorded for the control, non-manipulated session (CAMK2A sessions n = 8, VGAT sessions n = 12); 1648 CAMK2A and 784 VGAT neurons were recorded for the rotated session (CAMK2A sessions n = 8, VGAT sessions n = 11). Place cell analysis was performed separately on the first and second session halves, for both the rotated and control sessions (Supplemental Tables 2–3). Consistent with previous reports, CAMK2A cells showed a significant reorientation of place field centers, as is qualitatively apparent in Fig. 4B (left) where the place field centers remain stable for the control session but appear to rotate by 180 degrees in the rotated session. In contrast, while VGAT cells similarly appear to maintain their place field centers across control session halves, indicating a degree of within-session stability, they did not systematically rotate their centers for the second half of the flipped session (Fig. 4B, right). This difference is quantified in Fig. 4D; while both cell types show a significant decrease in r-values for the rotated session (Supplemental Fig. 2), significantly more CAMK2A than VGAT cells rotated their place field centers from one outer-third of the enclosure to the other during the flipped session. Place cells with fields in the middle third were excluded from this analysis, as it is not possible to determine whether these cells rotate their place fields. (O1 → O2, 55.1% of CAMK2A place cells rotated their place fields as compared to 41.1% of VGAT place cells; Fig. 4C,D). Moreover, CAMK2A cells were more stable than VGAT cells during the control session, as significantly more cells maintain their place field centers in the same outer third of the enclosure with no environmental perturbation (O1 → O1, 72.5% of CAMK2A place cells maintained their place fields as compared to 55.2% of VGAT place cells), and both CAMK2A and VGAT cells showed a significant increase in the proportion of cells that rotated from control to rotated sessions (Fig. 4D). Thus, VGAT cells responded to this environmental perturbation in a markedly different manner than the previously-described CAMK2A population. While there was a general increase in the number of place fields and decrease in mutual information following environmental manipulation, there was no significant difference in these metrics between the place cells that did and did not rotate their place field centers (Fig. 4, Supplemental Fig. 3). Finally, while CAMK2A cells were more likely to remain stable in the control session (‘Stable’ cells were defined as place cells that maintained their place field centers in the same third of the enclosure across session halves) and shift their place fields in the rotation session, this does not appear to be an inherent property that was maintained across sessions for individual place cells; comparing the activity of coregistered place cells across control and rotation sessions, the numbers of stable and unstable cells in the control session that rotated or did not rotate their place field in the experimental session were not significantly different (Fisher’s Exact Test; CAMK2A n = 187; VGAT n = 32).

**Figure 5:**
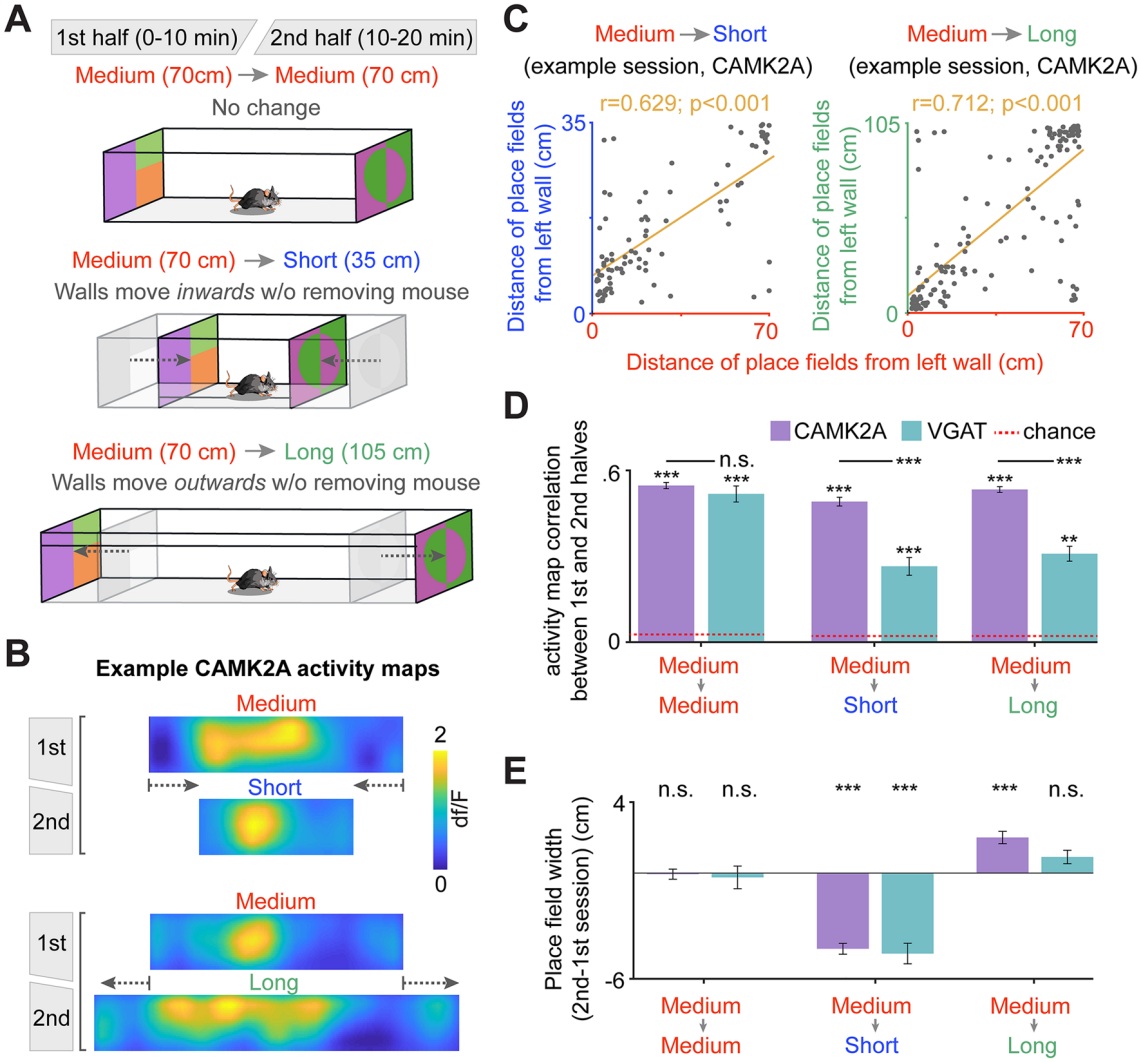
CA1 CAMK2A place cells bidirectionally scale place field size if the environment is shortened or lengthened, while VGAT cells only show place field contraction during environmental shortening. (**A**) Mice were placed in a box with moveable walls that allow for the maintenance (top, medium → medium), contraction (middle, medium → short) or lengthening of the environment (bottom, medium → long). The mouse was not removed from the environment while the walls were moved. Ten minutes of activity were recorded in each size. The original length of the environment was 70 cm, and it was either shortened or lengthened by 50%, or 35 cm, resulting in lengths of 35 or 105 cm. The width and height of the box were not changed. (**B**) Example CAMK2A activity maps. Note that the relative position of the place field is maintained before and after changing the environment’s length. In these examples, the place field size contracts and expands following shortening and lengthening of the environment, respectively. (**C**) Correlation of place field center location for all place cells in the first recording half following environment shortening (left) and lengthening (right) for a representative mouse with recordings of CAMK2A cells (Spearman correlation; medium → short cell n = 89, medium → long cell n = 154 from single example mouse). (**D**) Bars show the rate map correlation across different environmental length changes for all cells categorized as place cells in the first session half (mean correlations: CAMK2A: medium → medium = 0.55 ± 0.01, medium → short = 0.49 ± 0.02, medium → long = 0.53 ± 0.01; VGAT: medium → medium = 0.51 ± 0.03, medium → short = 0.26 ± 0.03, medium → long = 0.31 ± 0.03). Chance levels of correlation are shown by the red dashed line (see “Methods”). A cell was included in this analysis if it was categorized as a place cell in the first session half. [Statistics and sample sizes: Wilcoxon signed-rank test for within-session comparisons; medium → medium CAMK2A z = 21.56, VGAT z = 10.50; medium → short CAMK2A z = 18.27, VGAT z = 6.95; CAMK2A medium → long z = 23.19, VGAT z = 8.45; Wilcoxon rank-sum test for between-session comparisons; medium → medium CAMK2A/VGAT z = − 0.52; medium → short CAMK2A/VGAT z = 6.31; medium → long CAMK2A/VGAT z = 8.05; medium → medium CAMK2A n = 658 (from 8 mice), VGAT n = 182 (from 12 mice); medium → short CAMK2A n = 544 (from 8 mice), VGAT n = 213 (from 12 mice); medium → long CAMK2A n = 763 (from 8 mice), VGAT n = 169 (from 12 mice)] (**E**) Quantification of place field width change (second session half—first session half). CAMK2A place field width showed no change in medium → medium sessions (− 0.06 ± 0.29 cm), contraction for medium → short sessions (− 4.30 ± 0.31 cm) and an increase for medium → long sessions (2.02 ± 0.35 cm). VGAT place fields did not show consistent size changes in medium → medium sessions (− 0.24 ± 0.64 cm) or medium → short sessions (mean 0.91 ± 0.38 cm), but showed contractions in medium → short sessions (− 4.57 ± 0.59 cm). A cell was included in this analysis only if it was independently categorized as a place cell in each session half. [Wilcoxon signed-rank test; medium → medium CAMK2A n = 244 (from 8 mice), z = 0.17, VGAT n = 49 (from 12 mice), z = − 0.22; medium → short CAMK2A n = 166 (from 8 mice), z = − 8.42, VGAT n = 46 (from 12 mice), z = − 4.03; medium → long CAMK2A n = 256 (from 8 mice), z = 5.93, VGAT n = 32 (from 12 mice)] ***p < 0.001, **p < 0.01.

We next tested another environmental manipulation whose effects have been previously reported for CAMK2A neurons; instead of rotating the proximal cues halfway through the session, we rather increased or decreased the enclosure length by 50% to determine if VGAT cells would rescale their place fields to the same degree observed of CAMK2A neurons (Fig. 5A; see ‘Short-long linear track’ section in “Methods”)^2–4^. A total of 2281 CAMK2A and 946 VGAT neurons were recorded for the medium to medium session (medium → medium; CAMK2A sessions n = 8, VGAT sessions n = 12); 2048 CAMK2A and 799 VGAT neurons were recorded for the medium to short session (medium → short; CAMK2A sessions n = 8, VGAT sessions n = 11); 1822 CAMK2A and 830 VGAT neurons were recorded for the medium to long session (medium → long; CAMK2A sessions n = 8, VGAT sessions n = 11) (Supplemental Tables 4–6). In agreement with previous reports, many CAMK2A place cells contracted their place fields for the medium → short manipulation and expanded them for the medium → long manipulation (Fig. 5B). For an example medium → short and medium → long session of a single CAMK2A cell recording, the place field center locations were significantly correlated between session halves, indicating that the relative location was preserved by single cells across the experimental manipulation (Fig. 5C). Across all sessions, the activity maps of CAMK2A and VGAT cells were more correlated than expected by chance (Fig. 5D; ‘chance’ is denoted by the dotted red line). However, while CAMK2A and VGAT place cell activity maps were similarly correlated across medium → medium session halves, CAMK2A cells exhibited a significantly greater correlation than VGAT cells for the medium → short and medium → long assays, indicating this cell type exhibits a stronger rescaling tendency than VGAT cells. This was further evident when comparing the place field width of these cell types across rescaling assays. While place field width is unchanged across the control medium → medium assay, the width significantly decreases for both cell types in the medium → short assay and increases for only CAMK2A cells in the medium → long assay (Fig. 5E). Taken together, these results suggest that VGAT cells do not rescale their place fields to the same degree as has been reported for CAMK2A neurons.

**Figure 6:**
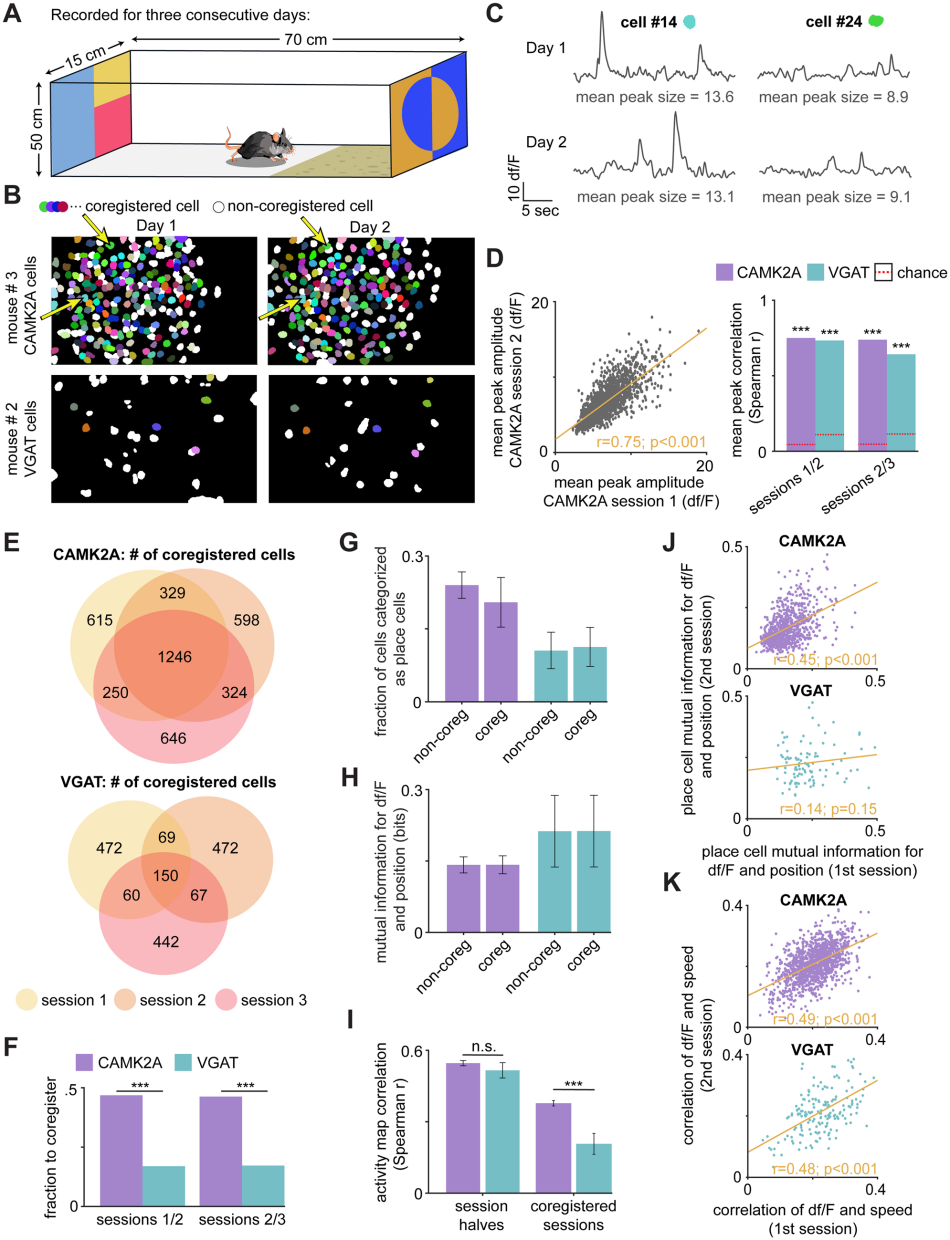
CAMK2A cells are more likely than VGAT cells to be co-active across days. (**A**) Scheme showing the box in which recordings were obtained. (**B**) Example spatial contours of CAMK2A cells identified in day 1 and day 2 (top row). White contours represent cells that were found only in one session (non-coregistered), while colorful contours represent cells that were co-registered across both sessions. Cells in the same location with the same color in both plots were identified as being the same cell (i.e., co-registered) in both recording sessions, separated by 24 h. The bottom row shows the same as the top row, but for VGAT cells. Note that, relative to CAMK2A cells, a smaller fraction of VGAT cells are co-active and co-registered across 2 days in these representative examples. (**C**) Shown are df/F traces from two example CAMK2A cells (annotated by arrows in (**B**) that were coregistered across days 1 and 2. The mean peak amplitude for coregistered cells is similar across sessions. (**D**) (left) Spearman correlation of mean peak amplitude from an example session for all co-registered cells between days 1 and 2. Note that the mean peak amplitude is highly correlated across sessions, indicating it is a stable feature of cell activity across days. (right) Bars show the correlation of mean peak amplitude for all coregistered cells, CAMK2A and VGAT, between sessions 1/2, and sessions 2/3 (CAMK2A correlation: 1/2 = 0.74, 2/3 = 0.73; VGAT correlation: 1/2 = 0.78, 2/3 = 0.69). The dotted red lines represent chance, or the upper 95th percentile of the shuffled distribution. (Statistics and sample sizes: Spearman correlation; CAMK2A sessions n = 7, VGAT sessions n = 8; sessions 1/2: CAMK2A n = 1575, VGAT n = 219; sessions 2/3: CAMK2A n = 1570, VGAT n = 215). (**E**) Venn diagram depicting the number of co-registered cells across 3 sessions for CAMK2A (top) and VGAT cells (bottom) (For each recording day, CAMK2A sessions n = 7, VGAT sessions n = 8; CAMK2A session 1 n = 2240, session 2 n = 2466, session 3 n = 2497; VGAT session 1 n = 751, session 2 n = 758, session 3 n = 719). (**F**) Bars show the fraction of cells that were co-registered between sessions 1/2 and 2/3 for CAMK2A and VGAT mice (CAMK2A: 1/2 = 0.47, 2/3 = 0.46; VGAT: 1/2 = 0.16, 2/3 = 0.17). A greater fraction of CAMK2A than VGAT cells were coregistered across session pairs. (Statistics and sample sizes: Fisher’s exact test; sessions 1/2: CAMK2A coregistered n = 1575, CAMK2A non-coregistered n = 1787, VGAT coregistered n = 119, VGAT non-coregistered n = 1190; sessions 2/3: CAMK2A coregistered n = 1570, CAMK2A non-coregistered n = 1823, VGAT coregistered n = 217, VGAT non-coregistered n = 1043; CAMK2A sessions n = 7, VGAT sessions n = 8). (**G**) Bars depict the fraction of cells to be categorized as place cells, separately for cells that coregistered and did not coregister across days (non-coregistered: CAMK2A = 0.24 ± 0.03, VGAT = 0.11 ± 0.04; coregistered: CAMK2A = 0.20 ± 0.05, VGAT = 0.11 ± 0.04; CAMK2A n = 7; VGAT n = 8). (**H**) Bars depict the mutual information between df/F and position, separately for cells that coregistered and did not coregister across days (non-coregistered: CAMK2A = 0.14 ± 0.02, VGAT = 0.21 ± 0.07; coregistered: CAMK2A = 0.14 ± 0.02, VGAT = 0.22 ± 0.08; CAMK2A n = 547/1681; VGAT n = 440/258; CAMK2A sessions n = 7, VGAT sessions n = 8). (**I**) Bars show the Spearman correlation of rate maps flattened across the length of the enclosure, for CAMK2A and VGAT cells across session halves (left; Statistics and sample sizes: Wilcoxon rank sum test; CAMK2A n = 650; VGAT n = 146, z = − 0.68, CAMK2A sessions n = 7, VGAT sessions n = 8) as well as those that coregistered across session pairs (right; Statistics and sample sizes: Wilcoxon rank sum test; CAMK2A n = 940; VGAT n = 108, z = 5.69; CAMK2A sessions n = 7, VGAT sessions n = 8). (Session half mean r-values: CAMK2A = 0.55 ± 0.01, VGAT = 0.52 ± 0.03; coregistered session mean r-values: CAMK2A = 0.37 ± 0.01, VGAT = 0.20 ± 0.04), (**J**) Scatterplots show the mutual information between df/F and position for CAMK2A (top) and VGAT (bottom) cells that coregistered across session pairs (Spearman correlation; CAMK2A n = 940; VGAT n = 108). (**K**) Scatterplots show the correlation of Spearman r-values between df/F and speed for CAMK2A (top) and VGAT (bottom) cohorts across coregistered sessions recorded 24 h apart (Spearman correlation; CAMK2A n = 1259; VGAT n = 172). ***p < 0.001.

Having examined the within-session correlation of CAMK2A and VGAT cell activity maps, we next studied the across-session stability of these cell types to determine if the same neurons were active during exploration on contiguous recording days. To do so, we analyzed the calcium recordings from habituation days 1–3 of the context rotation assay (Fig. 6A; see Supplemental Table 7 for cell counts). Cells were coregistered across days by the similarity of their spatial footprints using CellReg, an open-source probabilistic modeling package^52^ (see Supplemental Figs. 4 and 5 for maximum projection images of all coregistered sessions). As is qualitatively apparent from the examples shown in Fig. 6B, a greater proportion of CAMK2A than VGAT cells were coregistered across days (see Supplemental Fig. 6 for individual examples of coregistered VGAT cells). To determine if this was due to a fault in the coregistration procedure, we reasoned that the same cells would exhibit similar calcium peak amplitudes across days. Thus, to validate the coregistration results, we further compared the mean peak amplitude of CAMK2A and VGAT cells across sessions and found this measure to be strongly correlated for each cell type (Fig. 6C,D). The same is true of the coregistered peak-to-noise ratios across recording sessions (Supplemental Fig. 7). Thus, with similar spatial footprints and highly correlated peak amplitude and peak-to-noise ratio, the coregistration results are similarly accurate for both cell types. We next quantified this difference in coregistration across sessions. The Venn diagrams in Fig. 6E, which show the overlap in coregistered cell counts for all mice across the three recording sessions, indicate a much greater proportion of CAMK2A than VGAT cells were observed across all three sessions. Indeed, a significantly greater proportion of CAMK2A than VGAT cells were coregistered between session pairs 1/2 and 2/3 (Fig. 6F; CAMK2A: 1/2 = 0.47, 2/3 = 0.46; VGAT: 1/2 = 0.16, 2/3 = 0.17), suggesting that CAMK2A cells are more stable than VGAT cells across days. Comparing the encoding properties of cells that were active and inactive across sessions (i.e. those that coregistered or did not coregister), we found no significant difference in the fraction of cells that were categorized as place cells (Fig. 6G) or the mutual information for df/F and spatial position (Fig. 6H); thus, neither CAMK2A nor VGAT stability appears to predict the degree to which these cells encode spatial location. However, we did find that place encoding metrics remain far more stable across sessions for CAMK2A than VGAT cells. While activity maps show strong correlation for both cell types across session halves, CAMK2A activity maps correlate significantly more than VGAT maps across days (Fig. 6I). Further supporting this view, place cell mutual information for df/F and position is highly correlated across sessions for only the CAMK2A group (Fig. 6J). Speed encoding, on the other hand, showed greater pan neuronal stability and was maintained across session halves for both cell types (Fig. 6K).

## Discussion

We showed that compared to CAMK2A cells, VGAT place cells exhibited smaller place field size and higher mutual information between activity and location (Fig. 2), indicating this subset may encode spatial location to a more specific degree. Additionally, large subsets of both cell types encoded speed (Fig. 3).

As previously reported, CAMK2A cells remapped in a predictable manner following two different environmental manipulations—a rotated linear track (Fig. 4) and a short-long linear track (Fig. 5)—respectively rotating or rescaling their place fields with the enclosure. The effects of such manipulations have never, to our knowledge, been analyzed for CA1 GABAergic cells or directly compared with their pyramidal neighbors; notably, these cells exhibited fewer place field rotations and lower activity map correlation, pre- versus post-manipulation than CAMK2A cells.

Lastly, VGAT cells showed marked instability and were less likely than CAMK2A cells to be active across days. This instability extended to their encoding of place; in a novel across-day comparison, we also show that VGAT cells were also less likely to maintain their spatial encoding across days (Fig. 6). Taken together, these data show both CAMK2A and VGAT CA1 cells encode variables relevant for navigation, though with key differences.

The present work has made two main novel contributions to the existing interneuronal place cell literature. (1) To our knowledge, there have been no reports to date on the effects of spatial perturbations on interneurons. Such observations are crucial in building more accurate models of the features and constraints of hippocampal dynamics in spatial coding. We therefore replicated pyramidal place cell analyses for interneurons, pre- and post-rotational or rescaling manipulations. In each case, pyramidal cells reoriented their fields to rotations and re-sizing to a greater degree than VGAT cells (Figs. 4D, 5D,E). Thus, in general, CA1 interneuron place cells appear to both represent space to a finer degree than pyramidal neurons, yet exhibit less flexibility and stereotyped shifts to environmental change. It is possible these interneurons encode distal cues to a greater degree than pyramidal cells, resulting in less place field rotation, though further experimentation would be necessary to verify this hypothesis. (2) We show that interneurons are less likely to both remain active across days (Fig. 6E,F) and exhibit lower consistency of place encoding across consecutive recording days (Fig. 6I,J) than neighboring pyramidal cells.

### Considering the application of calcium imaging to interneuronal recording

Prior reports indicated that numerous interneuronal subtypes exhibit high baseline firing rates^21–24,43^. Though calcium imaging has been extensively used to analyze interneuronal activity, both within the hippocampus^25,35–41^ and cortex^53–62^, it is important to consider that this method may not fully capture the temporal dynamics of VGAT interneurons with high firing rates. Nevertheless, calcium imaging has been successfully used to target a wide variety of interneuron groups in the hippocampus including bistratified cells^25^, vasoactive intestinal peptide-expressing interneurons^36,37,39,41^, cck-expressing interneurons^40^ and fast-spiking parvalbumin-expressing interneurons^25,38,40^; these data have been used in analyses that required a high degree of temporal precision, such as the estimation of spike timing in relation to theta rhythm^35^, as well as analyses which, similar to the present study, necessitated the comparison of cell types with differing baseline firing rates^38^. Furthermore, it is crucial to note that, using calcium imaging, we have replicated key prior findings obtained with electrode recordings, including higher baseline activity (Fig. 1H)^21–24,43^, higher mutual information between neural activity and location (Fig. 2E)^21,22^, and stronger encoding of speed by interneurons than pyramidal cells (Fig. 3G)^33,34^. Taken together, this indicates that calcium imaging can be used to gain insight on interneuronal function, producing comparable results to those obtained from electrode recordings.

Moreover, it is unlikely that the difference in temporal dynamics between cell types could account for our most novel findings concerning place cell stability across days and spatial manipulations, as the absolute value of cell type-specific firing rates is not critical in these analyses. For instance, it is intuitive that fast-spiking interneurons should be identified from raw miniscope recordings with a greater probability than sparsely-firing pyramidal cells; to the contrary, we found that interneurons were less likely to be active across recording sessions, indicating that the difference in baseline firing rate did not bias this novel result. Indeed, due to electrode drift, this type of across-day analysis necessitates the use of calcium imaging, and cannot be reliably done with electrodes.

### Comparison with other CA1 speed encoding studies

It has been shown that CA1 cells exhibit speed-correlated spiking activity^25–32,63,64^. A prior electrophysiological study^33^ found the CA1 speed cell population to be composed exclusively of interneurons; their analyses suggested that the activity of interneurons, not pyramidal cells, could predict animal speed. In contrast, we could predict animal speed to a sub-second timescale and with comparable accuracy using the activity of either CAMK2A or VGAT cells (Fig. 3F,G). While more VGAT cells showed greater positive correlation with speed in our data (Fig. 3C) and decoding accuracy was slightly higher for VGAT than CAMK2A cells, our analyses indicate that both cell types encode speed to a significant degree (Fig. 3G).

Leveraging the advantages afforded by calcium imaging, we were able to track speed encoding across days in the same cells. We thus show that speed encoding for GABAergic cells is maintained across days, even though these same cells show significant remapping of spatial encoding (Fig. 6I–K).

### Discriminating CA1 interneuronal subtypes

The CA1 pyramidal layer, which we surgically targeted to image cell activity, houses several interneuronal classes including axo-axonic chandelier cells, parvalbumin and cholecystokinin basket cells, bistratified cells, and nitric oxide synthase-expressing ivy cells, among others^65,66^. It is important to note that these slow spiking ivy cells are the most well-represented interneuronal class of the pyramidal layer, and likely constitute the largest proportion of recorded VGAT neurons in this study^66^. Previous work suggests that there is an encoding overlap amongst interneuron subtypes for both speed and spatial location. In a two-photon calcium imaging study of parvalbumin- and somatostatin-expressing interneurons in CA1^38^, report that 14% of parvalbumin interneurons and 19% of somatostatin interneurons showed activity increases during immobility, closely resembling the 17% observed in our VGAT dataset (Fig. 3C) that showed a similar relationship. These negatively-correlated cells were anatomically heterogeneous and distributed across both the stratum oriens and stratum pyramidale layers of CA1^38^. Additionally, it was recently reported that CCK-expressing basket cells show similar immobility-aligned activity increases, exhibiting spike rates that are inversely proportional to those of CA1 pyramidal cells^40^. Another recent study reported that the layer-specific location within the hippocampus, rather than subtype, predicted whether interneurons were positively or negatively correlated with speed; moreover, spatial selectivity was observed in all identified interneuron subtypes^25^. Thus, it is likely the speed and place encoding interneurons we observed are also composed of a diverse mix of interneurons. Future studies may be able to further differentiate the diverse CA1 interneuronal population, as shown previously^25,38^, to contrast these characteristics and aid in more accurate models of CA1 function.

In summary, here we characterize for the first time the relative stability of CA1 interneurons and pyramidal cells, both in relation to spatial coding across environmental rotation and rescaling and the consistency of activity across days, providing a more nuanced picture of the parameters that constrain or shift the hippocampal network in a dynamic environment.

## Methods

All procedures conformed to guidelines established by the National Institutes of Health and have been approved by the University of California, Los Angeles Institutional Animal Care and Use Committee, protocols 2017-011 and 2017-075.

### Mice

VGAT-Cre mice (Jackson Laboratory stock No. 028862) were used for all experiments. Male and female mice between 2 and 6 months of age were used in all experiments. Mice were maintained on a 12-h reverse light–dark cycle with food and water ad libitum. All mice were handled for a minimum of 5 days prior to any behavioral task. VGAT-cre mice were used to direct the GCaMP6f expression in GABAergic neurons, since VGAT, or vesicular GABA transporter, is a gene specifically expressed in GABAergic inhibitory neurons. No animals were excluded. This study is reported in accordance with ARRIVE guidelines.

### Viral vectors

All vectors were purchased from Addgene.

### Surgeries

Surgical methods were similar to those reported elsewhere^67^. Mice were anesthetized with 1.5 to 2.0% isoflurane for surgical procedures and placed on the stereotaxic frame. Mice were unilaterally injected with 500 nl of either AAV9.CAMK2A.GCaMP6f. (Addgene #107790) or AAV9.DIO.EF1a.GCaMP6f (Addgene #128315) virus at 80 nl/min into the dorsal CA1 at the stereotactic coordinates − 2.1 mm posterior to bregma, 1.6 mm lateral to midline and − 1.60 mm ventral to skull surface. Mice were randomly allocated in each cage to receive either one of the viral vectors above. Following 4 days of recovery, a microendoscope was implanted above the injection site. The microendoscope (GRIN lens, 0.25 pitch, 0.50 NA, 1.8 mm in diameter, Edmund Optics) was placed above the CA1 region of the hippocampus by stereotaxic arm at a depth of 1.35 mm, ventral to the skull surface. A 2.0 mm diameter circular craniotomy was centered 0.5 mm medial to the virus injection site. The cortex directly below the craniotomy was aspirated with a 27-gauge blunt syringe needle attached to a vacuum pump exposing the hippocampal commissural fibers. Phosphate-buffered saline (PBS) was repeatedly applied to the exposed brain tissue to prevent drying. The microendoscope was slowly lowered with a stereotaxic arm above CA1 to a depth of 1.3 mm ventral to the surface of the skull. A skull screw was placed, and the lens was secured with cyanoacrylate glue and dental cement. The GRIN lens was protected with Kwik-seal glue and animals were returned to a clean cage. Two weeks later, a small aluminum base plate was cemented onto the animal’s head atop the previously formed dental cement.

### Perfusion and histological verification

Mice were anesthetized with Fatal-Plus and transcardially perfused with PBS, followed by a solution of 4% paraformaldehyde. Extracted brains were stored for 12 h at 4 °C in 4% paraformaldehyde. Brains were then placed in sucrose for a minimum of 24 h. Brains were sectioned in the coronal plane in a cryostat, washed in phosphate buffered saline and mounted on glass slides using PVA-DABCO. Images were acquired using a Keyence BZ-X fluorescence microscope with a 10 × or 20 × air objective.

### Behavioral protocols

(Measurements are written as length x width x height in cm). The authors that conducted each behavioral assay and the data analysis were blinded to group assignment (VGAT-GCaMP6f or CAMK2A-GCaMP6f).

#### Mice

The full CAMK2A and VGAT cohorts were, respectively, 8 and 12 mice. There were, however, miniscope recording issues that led to occasional dropouts, rendering the data from the affected sessions unusable. Thus, the total number of mice varied slightly in the following assays as fully documented in Supplemental Tables 1–7.

#### Assay timelin

(See individual assay descriptions below.)*:* days 1–3: Linear track; days 4–8: Rotated linear track; days 9–11: Short-long linear track.

#### Linear track

Mice were placed in an empty linear track (70 × 9 × 50 cm) for a period of 20 min over three consecutive days at 40 lx illumination. The first two days were habituation sessions and the third was used in the analysis in Figs. 2 and 3. To encourage the formation of stable place fields, local cues were provided in the form of colored tape along the walls of the enclosure (CAMK2A sessions n = 8, VGAT sessions n = 12).

#### Rotated linear track

One day after the linear track assay described above, mice were then placed in a different empty linear track (70 × 15 × 52 cm) over five consecutive days at 40 lx illumination. To encourage the formation of stable place fields, visual cues were positioned at the extremities, and, on one side, a visual and sensory cue was added to the floor. At the beginning of each session, mice were placed in the center of the linear track, always facing towards the left. The first three days were 10-min habituation sessions without rotation. Recordings from these 3 days were used in the across-session coregistration analysis in Fig. 6 (CAMK2A sessions n = 7, VGAT sessions n = 8).

During the 4th and 5th sessions in this assay, the linear track was rotated respectively either 0 or 180 degrees halfway through the session (10 min before and 10 min after rotation). Mice were removed from the enclosure between orientation changes in all sessions, both for 180 and control 0 degree rotations. This process took less than 30 s and the data acquired during this period was removed from the analysis. Data from the 4th session, which was done with 0 degree rotation, was named “orientation 1 → orientation 1” and used as the comparison control. The 5th session had a 180 degree rotation, and is referred to as “orientation 1 → orientation 2” in the Figures (non-rotation day: CAMK2A sessions n = 8, VGAT sessions n = 12; rotation day: CAMK2A sessions n = 8, VGAT sessions n = 11).

#### Short-long linear track

A linear track with adjustable length was constructed (maximum 105 × 8 × 52 cm) so mice could be exposed to three different lengths during the experiment: short (35 cm), medium (70 cm), and long (105 cm). Visual cues were placed at the extremities, illumination was maintained at 40 lx, and odor cues were used to differentiate this assay from the previous rotated linear track assay. At the beginning of each session, mice were placed in the center of the linear track, facing towards the left. Twenty-minute sessions were recorded for medium to medium (medium → medium), medium to short (medium → short) and medium to long (medium → long) recordings in separate days in this order. In the medium → medium control sessions, the length of the environment was maintained at 70 cm for the entire 20-min recording. For the medium → short recordings, the length of the environment was kept as the medium length for the first 10 min (i.e., the first half of the recording). Then, the track was shortened by 50% to 35 cm for the second half of the recording, which also lasted 10 min. This was accomplished by slowly moving both of the short walls inwards in full view of the mouse in a custom-built rescalable environment, as reported previously^3^. For the medium → medium control session the same noise and vibrations produced during length changing were produced, but without changing the context length. The data acquired during this brief interference with noise and vibrations was removed from the analysis of all sessions (For all short-long linear track sessions, CAMK2A sessions n = 8, VGAT sessions n = 12).

### Miniscope video capture

All videos were recorded at 30 frames/sec using a Logitech HD C310 webcam and custom-built head-mounted UCLA miniscope^67^. Open-source UCLA Miniscope software and hardware (http://miniscope.org/) were used to capture and synchronize the neural and behavioral videos^42,67^.

### Miniscope postprocessing

The open-source UCLA miniscope analysis package (https://github.com/daharoni/Miniscope_Analysis)^42^ was used to motion correct the miniscope videos. They were then temporally downsampled by a factor of four and spatially downsampled by a factor of two. The cell activity and footprints were extracted using the open-source package Constrained Nonnegative Matrix Factorization for microEndoscopic data (CNMF-E; https://github.com/zhoupc/CNMF_E)^44,68^. Only cells whose variance was greater than or equal to 10% of the maximum variance among non-outliers were used in the analysis^69–71^. Neurons were coregistered across sessions using the open-source probabilistic modeling package CellReg (https://github.com/zivlab/CellReg)^52^.

### Calcium peak rate and peak width metrics

The peak rate and width of each putative neuron were quantified by applying the Matlab function ‘findpeaks’ to the raw calcium trace of each cell.

### Behavioral quantification

To extract the pose of freely-behaving mice in the described assays, we implemented DeepLabCut^72^, an open-source convolutional neural network-based toolbox, to identify mouse nose, ear and tailbase xy-coordinates in each recorded video frame. These coordinates were then used to calculate speed and position at each time point. The position and speed vectors were smoothed by averaging over a 0.5 s moving window (Matlab function ‘smoothdata’).

### Activity maps

It has been demonstrated that CAMK2A and VGAT cells have grossly different firing properties^21–24,43^. To avoid systematically biasing the estimated activity of these cell types and to more accurately compare their encoding of behavioral variables^73^, we used the raw calcium traces output by CNMF-E for all analyses rather than deconvolved spike counts (e.g. by the OASIS algorithm^74^). Using the calcium activity directly, we also avoided any underlying assumptions of the deconvolution procedure. To calculate the activity map for each cell, we spatially binned the enclosure into 3.5 × 3.5 cm pixels and normalized by the total occupancy, resulting in the mean calcium activity of each neuron at each binned location. Only epochs for which both nose and tailbase speed exceeded 3 cm/s (periods of translational movement) were used in the place cell analysis. Activity maps were smoothed by convolution with a gaussian kernel (5 × 5 bins, 1 s.d. = 0.85 bins or 2.98 cm).

### Place cell properties

#### Mutual information and place cell classification

A standard information-theoretic approach^21,22,73,75^ was applied to the binned df/F and mouse position for each cell to determine its mutual information in bits. Only samples for which the mouse nose and tailbase speed exceeded 3 cm/s were used in this analysis:

$$I(X;Y) ={\sum }_{y\in Y} {\sum }_{x\in X} {p}_{\left(X,Y\right)}(x,y)log\left(\frac{{p}_{\left(X,Y\right)}(x,y)}{{p}_{X}(x) {p}_{Y}(y)}\right)$$

where *p(x)* is the binned distribution of mouse position and *p(y)* is the binned distribution of df/F. Sturges’ rule was used to determine the number of bins for df/F calcium data:

$$ {\text{Number }}\;{\text{of }}\;{\text{bins }} = { 1 } + {\text{ log}}_{{2}} n $$

Position information was binned at 3.5 cm increments across the length of the enclosure. To be classified as a place cell, a neuron’s mutual information had to exceed the 95th percentile of a bootstrapped distribution. To build this distribution, the calcium trace was circularly permuted 100 times by a constant increment (increment = the length of the recording / 100); the mutual information was calculated for each iteration.

#### Place field identification

Place fields were classified as contiguous activity map bins (connected by at least one bin side) that exceeded the mean df/F by more than 1.5 s.d.^68^ for cells that were classified as place cells. Please note that place cells could only have a discrete number of place fields. *Place field size* was quantified as the total area of a given place field. (place field size = area of a bin * # of contiguous bins) Similarly, *place field width* was quantified as the distance from end to end of a place field along the length of the enclosure.

### Speed cell classification

To categorize cells as significantly positively or negatively responsive to speed, the Spearman correlation was calculated for the raw calcium activity of each cell and mouse speed. This actual r-value was compared to a distribution of Spearman r-values built from circularly permuting the speed vector (100 iterations per session). Cells with an r-value greater or less than the top and bottom 95th percentile of this distribution were considered to be positively or negatively correlated with speed. In order to remove the possible influence of position on speed, a generalized linear model (GLM; Matlab function ‘glmfit’) was first used to model each cell’s activity by x-position. This modeled calcium activity was subtracted from the cell’s actual calcium activity.

### Speed decoding

To predict speed from calcium activity, the data was initially separated into alternating 60 s training and testing blocks, with 10 s of separation between blocks. Odd blocks were used to train a Generalized Linear Model (GLM) and withheld even blocks were used to test the resulting model. The GLM assigns weights to the activity of each neuron for the training data set; these weights are then applied to the activity of all neurons for the testing data set and used to predict the speed for held out samples. (Note that the predictor variables are the calcium activities of individual neurons, rather than the mean activity across neurons.) Accuracies of this withheld testing block were reported as the Spearman correlation between the actual and predicted behavioral metrics. The chance correlation level was calculated, per animal, as the top 95 percentile of a distribution of Spearman correlation scores built from circularly permuted data (100 iterations per session). As poor spatial sampling could affect GLM performance, mice whose path length fell 1 SD below the average path length were removed from the analysis. To address the possibility that differing numbers of recorded cells could affect decoding accuracy, GLMs were calculated by first subsampling the minimum number of cells across mice. (This subsampling was performed over 100 iterations and averaged.) Thus, all models were trained and tested using the same number of cells. We chose to train the GLM on the raw calcium signal, rather than deconvolved activity, because (1) using the calcium activity directly, we avoid any underlying assumptions of the deconvolution procedure^74^, (2) the slow decay of the calcium signal allows for neural activity preceding behavior to contribute towards the linear model of each behavioral metric, and (3) there is a convention to do so in the calcium imaging literature^76,77^.

### Activity map correlation

To determine the correlation of activity maps between session halves, the 2-dimensional activity maps were first flattened by taking the maximum bin at each point along the length of the enclosure. (Thus, a 3 × 20 matrix becomes a 1 × 20 vector.) The Spearman correlation coefficient was calculated for the resulting first and second session half activity vectors for the medium → medium sessions. To determine the Spearman correlation coefficient for enclosures of differing lengths (medium → short or medium → long), activity maps from the second session half were resampled (Matlab function ‘resample’) to match the length of the first session half. To determine the chance Spearman r-value, the activity maps from all VGAT and CAMK2A cells were concatenated across first and second session halves. The activity map vectors for the second halves were circularly shuffled over 1000 iterations and the Spearman r-value was calculated between first and second halves for each iteration, building a null distribution. ‘Chance’ was defined as the top 95th percentile of this null distribution.

### Population event classification

Calcium activity was deconvolved to find the calcium events for each neuron in a given session^74^; this multiunit activity was used to define population events as any sample for which the number of coactive cells exceeded the mean by 4 or more standard deviations^48,68^.

### Mean peak amplitude

To calculate the mean peak amplitude for a cell on a given session, the local maxima were identified from the raw calcium activity (Matlab function ‘findpeaks’), and these maxima amplitudes were averaged.

### Statistics

Nonparametric Wilcoxon signed-rank or rank-sum tests were used, unless otherwise stated using MATLAB. Two-tailed tests were used throughout with α = 0.05. Asterisks in the Figures indicate the *p* values. Standard error of the mean was plotted in each Figure as an estimate of variation. Multiple comparisons were adjusted with the false discovery rate (FDR) method.

## Supplementary Information

Supplementary Information.
