# Supplementary material for: GABAergic CA1 neurons are more stable following context changes than glutamatergic cells
Source: Sci Rep. 2022 Jun 20;12:10310. doi: 10.1038/s41598-022-13799-6 (PMC9209472; doi:10.1038/s41598-022-13799-6)
Supplement: Supplementary file 1 — Supplementary Information. [file 41598_2022_13799_MOESM1_ESM.docx]

**GABAergic CA1 neurons are more stable following context changes than glutamatergic cells**

**Peter J. Schuette^1,4^, Juliane M. Ikebara^2,4^, Sandra Maesta-Pereira^1^, Anita Torossian^1^ , Ekayana Sethi^1^ , Alexandre H. Kihara^2^, Jonathan C. Kao^3^, Fernando MCV Reis^1,5^, Avishek Adhikari^1,5,*^**

Affiliations

^1^ Department of Psychology, University of California, Los Angeles, Los Angeles, CA, 90095, USA.

^2^ Centro de Matemática, Computação e Cognição, Universidade Federal do ABC, SÃO Bernardo do Campo, SP, 09606-070, Brazil

^3^ Department of Electrical and Computer Engineering, University of California, Los Angeles, Los Angeles, CA, 90095, USA.

^4^ These authors contributed equally

^5^ These authors contributed equally

*Correspondence: avi@psych.ucla.edu

**Supplementary Information**

**
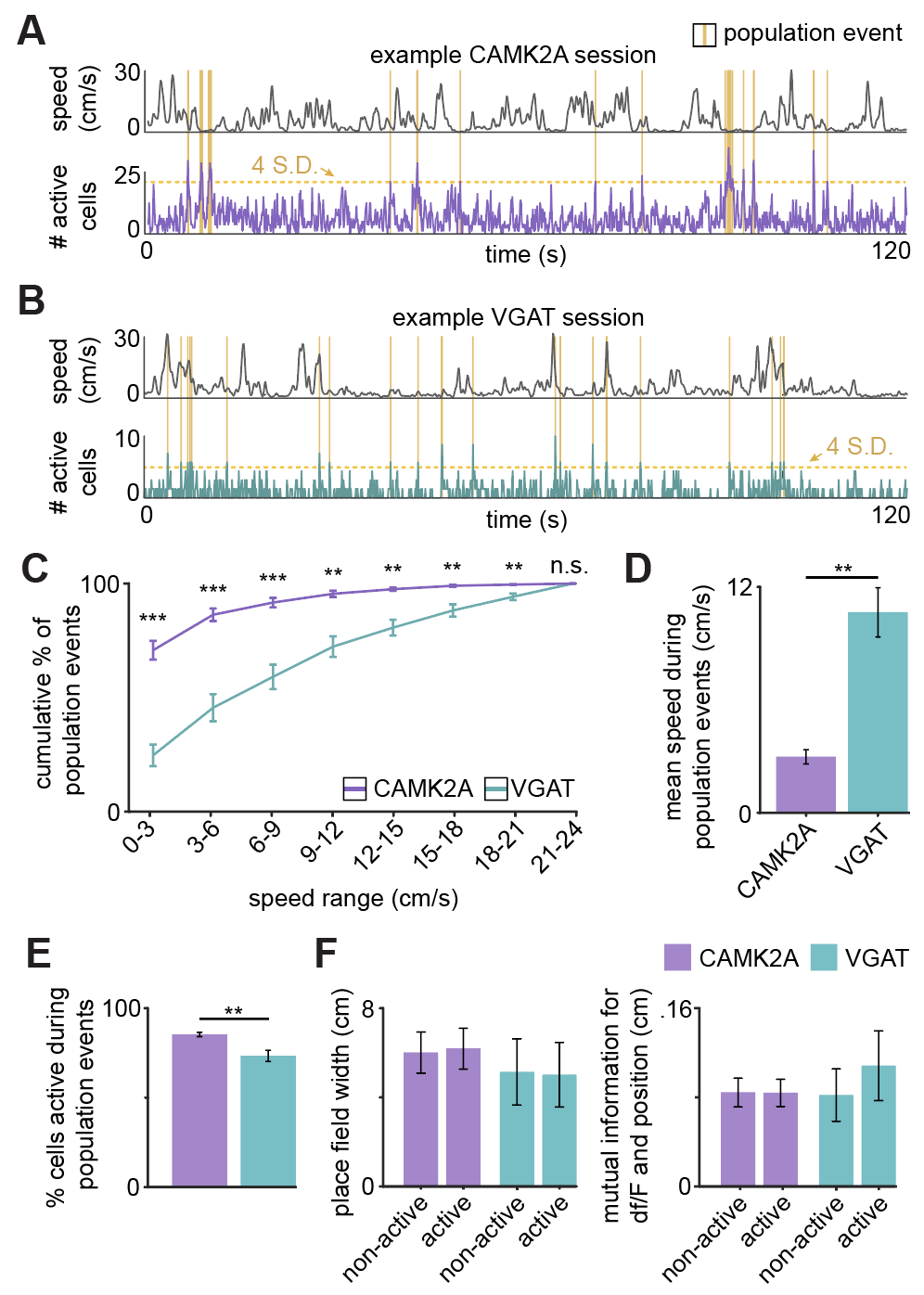
**

**Supplemental Figure 1.** The mean df/F activity of CAMK2A and VGAT cells increases, respectively, during low and high speed epochs. (**A**) Example data showing mouse speed (top, grey) and the mean z-scored df/F for CAMK2A cells (bottom, purple). Epochs in which the number of co-active cells exceeded 3 standard deviations (4 s.d. shown as yellow dashed line) were classified as “population events” and are marked with yellow vertical lines. Note that CAMK2A population events tended to occur during low speed epochs. (**B**) Same as (**A**), but for an example recording of VGAT cells. Note that VGAT population events tend to occur during high speed epochs. (**C**) Cumulative sum plot showing the distribution of population events for CAMK2A (purple) and VGAT (turquoise) cells across different speed ranges (two-sample t-test for each speed range comparison; CAMK2A sessions n=8, VGAT sessions n=12; for ascending speed ranges, t-statistic=7.71, 5.73, 4.85, 4.39, 4.15, 3.40, 3.00, 0). (**D**) Bars indicate that CAMK2A population events occur at lower average speed epochs (2.85±0.28 cm/s) relative to VGAT population events (7.02±0.74 cm/s; statistics and sample sizes: Wilcoxon rank sum test; n same as **C**; z=-3.20). (**E**) Bars indicate the percentage of CAMK2A and VGAT cells that were active during at least one population event (% CAMK2A=85.3±0.01, % VGAT=73.3±0.03) (statistics and sample sizes: Wilcoxon rank sum test; CAMK2A n=8; VGAT n=12; z=2.12). (**F**) Bars compare the (left) place field width and (right) mutual information between df/F and position for CAMK2A and VGAT cells that were either active or not active during population events (place field width (left): CAMK2A non-population events=6.01±0.92 cm, population events=6.18±0.92 cm; VGAT non-population events=5.14±1.48 cm, population events=5.01±1.45 cm; mutual information (right): CAMK2A non-population events=0.08±0.01 bits, population events=0.08±0.01 bits; VGAT non-population events=0.08±0.02 bits, population events=0.11±0.03 bits) (sample sizes: CAMK2A n=59/2252 (from 8 mice); VGAT n=108/848 (from 12 mice)). *** p<0.001, ** p<0.01.


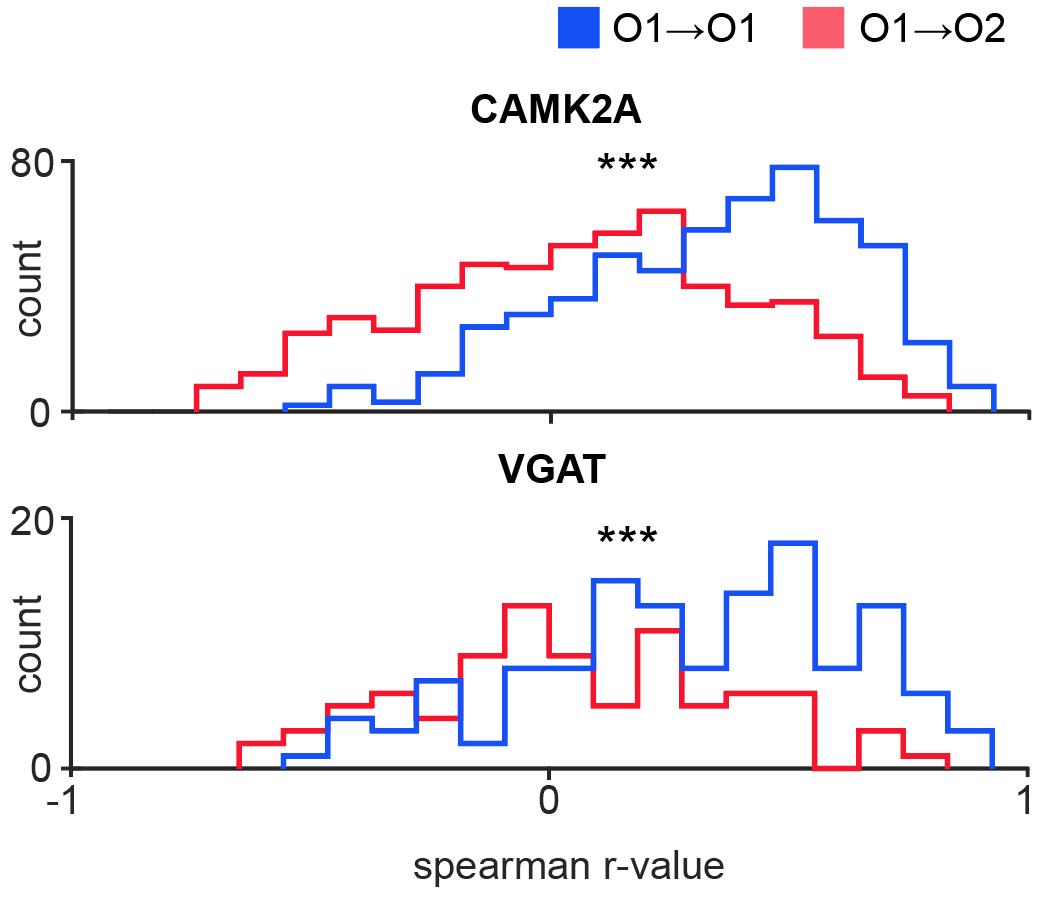


**Supplemental Figure 2.** Histograms depict the binned correlation counts of the activity maps of all place cells across session halves, for both the orientation 1-to-1 (blue) and orientation 1-to-2 sessions (red). (statistics and sample sizes: Wilcoxon rank sum test; CAMK2A orientation 1-to-1 n = 563, CAMK2A orientation 1-to-2 n = 561, z=-13.27, VGAT orientation 1-to-1 n = 137, VGAT orientation 1-to-2 n = 99, z=-5.28.) *** p<0.001.


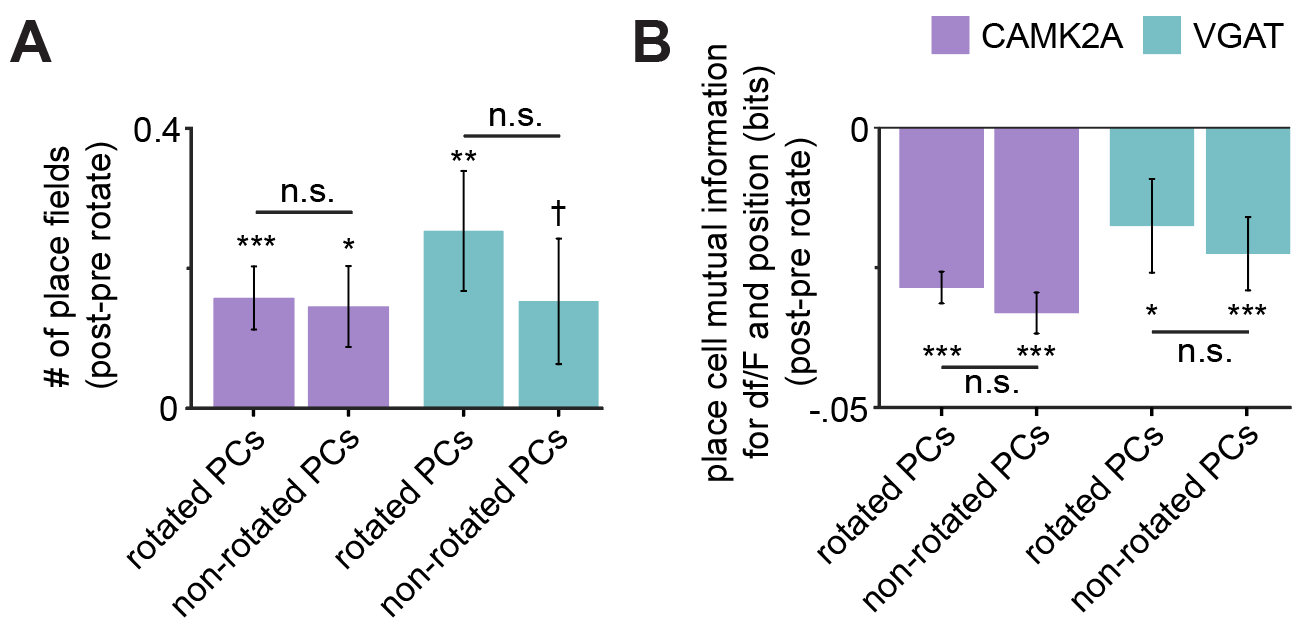


**Supplemental Figure 3.** Both CAMK2A and VGAT place cells exhibit an increase in number of place fields and a reduction in mutual information following an environmental rotation. (**A**) Bars represent the change in the number of place fields (post-pre rotation) for CAMK2A and VGAT place cells that either rotated or did not rotate their place fields (place field count difference: CAMK2A rotated=0.16±0.05, CAMK2A non-rotated=0.14±0.06; VGAT rotated=0.25±0.09, VGAT non-rotated=0.15±0.09) (statistics and sample sizes: Wilcoxon sign rank and rank sum tests; CAMK2A rotate n=261 (z=3.38), non-rotate n=145 (z=2.46); VGAT rotate n=67 (z=2.44), non-rotate n=72 (z=1.59)). (**B**) Similar to (**A**) except that the bars show the mutual information between neural activity and spatial location (post-pre rotation; mutual information difference: CAMK2A rotated=-0.03±0.003 bits, CAMK2A non-rotated=0.04±0.004 bits, VGAT rotated=-0.02±0.008 bits, VGAT non-rotated=-0.02±0.007 bits) (statistics and sample sizes: Wilcoxon sign rank and rank sum tests; CAMK2A rotate n=261, z=-4.39; non-rotate n=145, z=4.50; VGAT rotate n=67, z=-2.57; non-rotate n=72, z=-3.48). *** p<0.001. ** p<0.01, * p<0.05, † p=0.094.

**
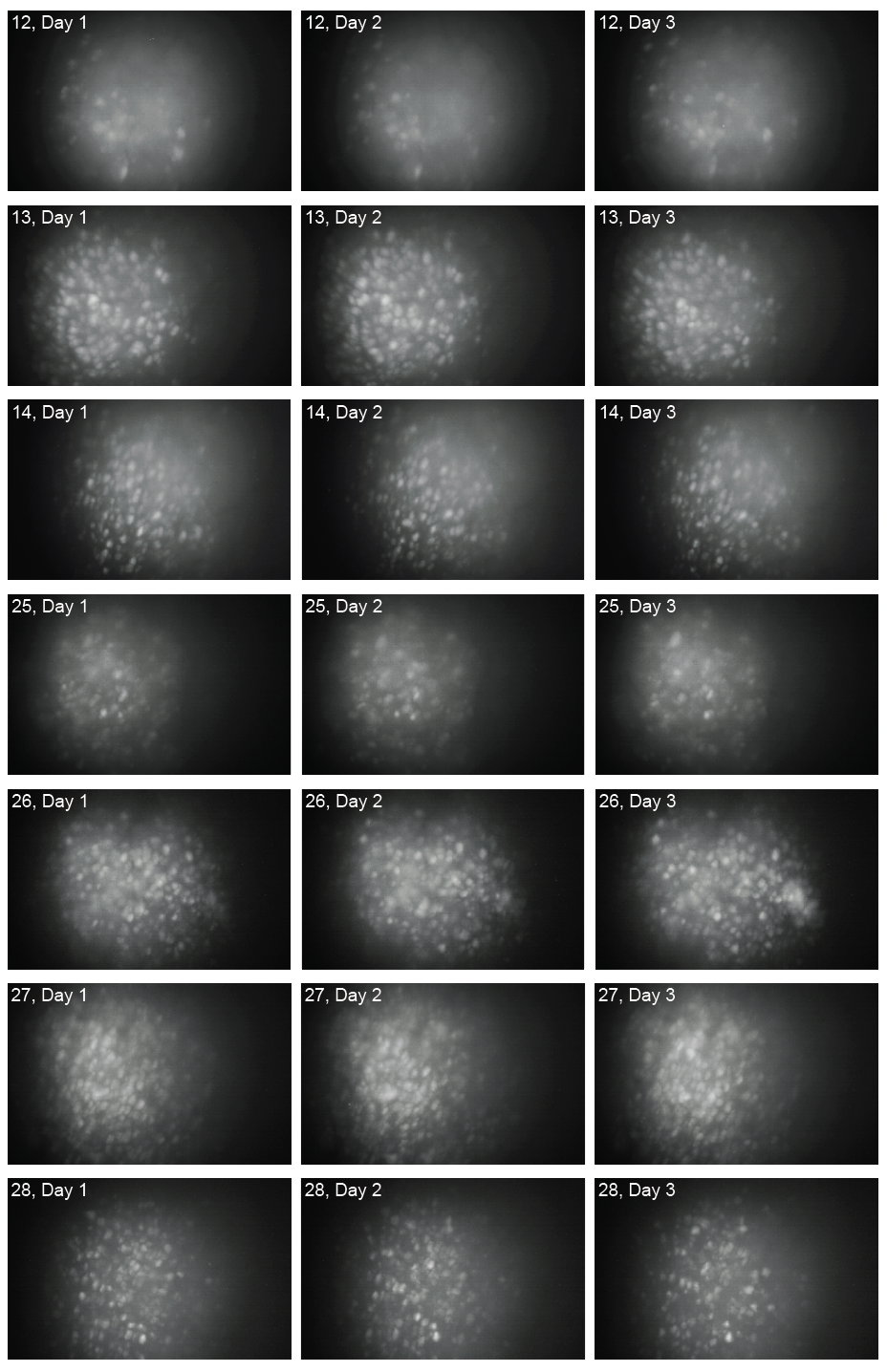
**

**Supplemental Figure 4.** Shown are the maximum projection images for all coregistered CAMK2A recordings.


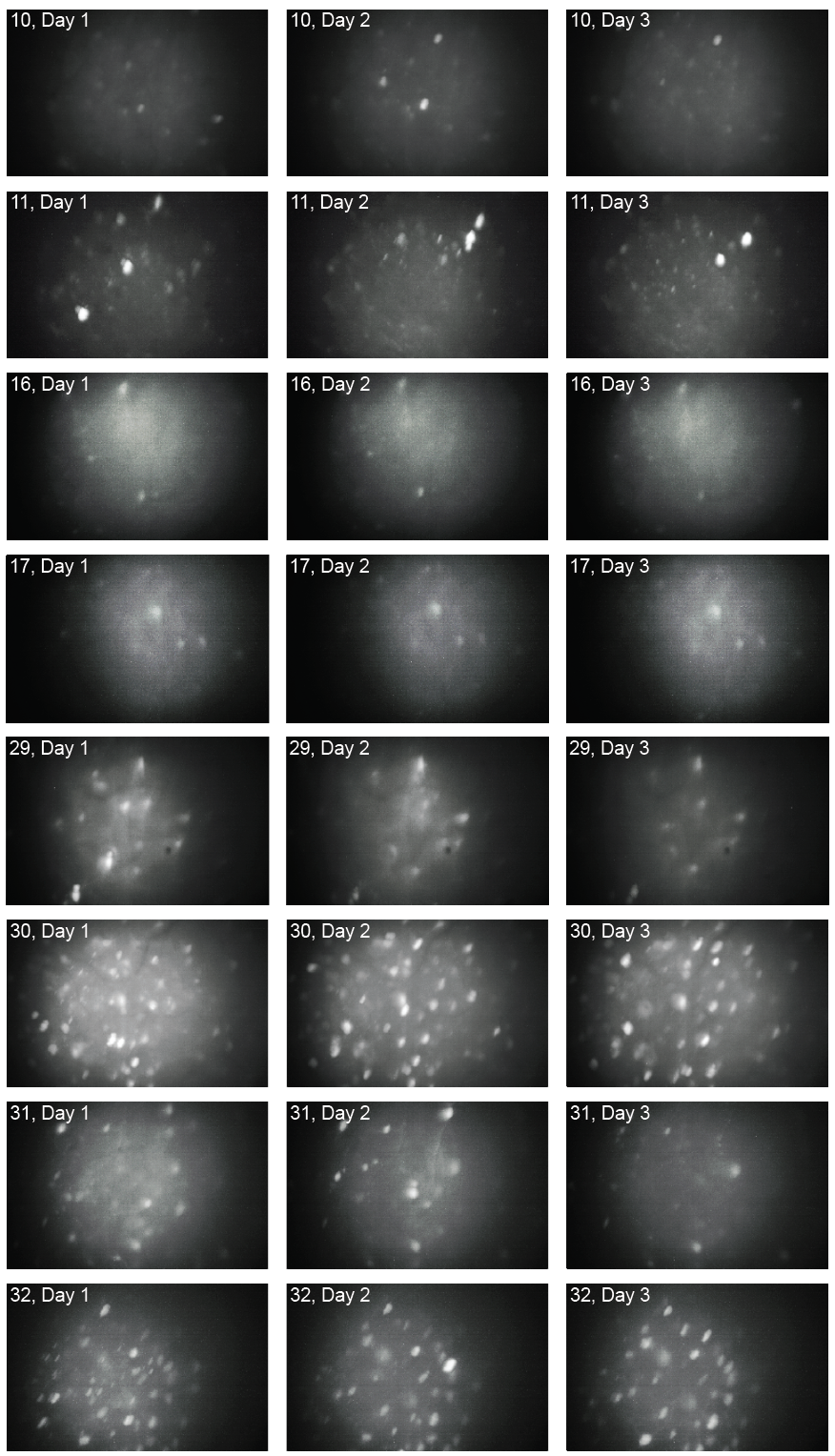


**Supplemental Figure 5.** Shown are the maximum projection images for all coregistered VGAT recordings.


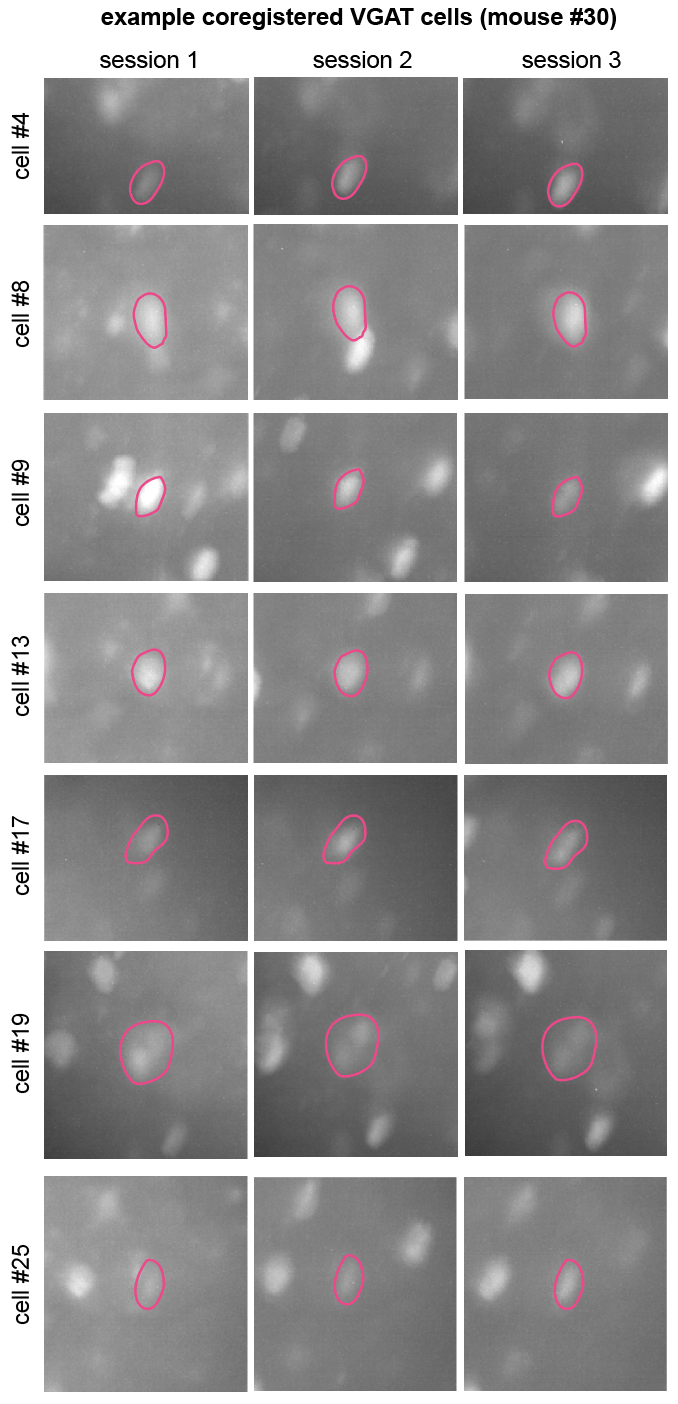


**Supplemental Figure 6.** Shown are zoomed-in maximum projection images from example mouse #30 with coregistered VGAT cells outlined in red for each recording session.


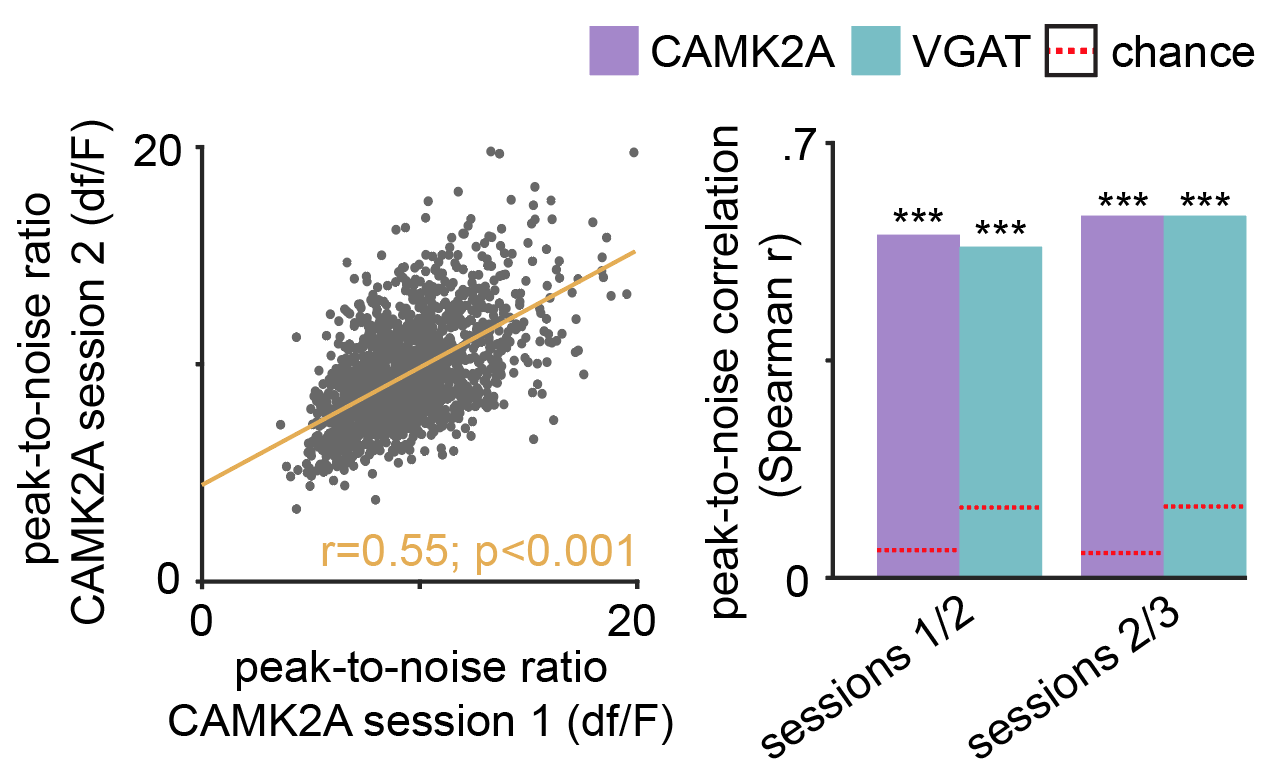


**Supplemental Figure 7.** (left) Spearman correlation of peak-to-noise ratio from an example session for all co-registered cells between days 1 and 2. Note that the peak-to-noise ratio is highly correlated across sessions, indicating it is a stable feature of cell activity across days. (right) Bars show the correlation of the peak-to-noise ratio for all coregistered cells, CAMK2A and VGAT, between sessions 1/2, and sessions 2/3 (CAMK2A correlation: 1/2=0.55, 2/3=0.58; VGAT correlation: 1/2=0.53, 2/3=0.58). The dotted red lines represent chance, or the upper 95th percentile of the shuffled distribution. (Statistics and sample sizes: Spearman correlation; CAMK2A sessions n=7, VGAT sessions n=8; sessions 1/2: CAMK2A n=1575, VGAT n=219; sessions 2/3: CAMK2A n=1570, VGAT n=215).

**Supplementary Tables**

**Linear Track (CAMK2A n=8, VGAT n=12)**

| **Mouse #** | **Cell Type** | **Cell count/Place cell count** |
| --- | --- | --- |
| 12 | CAMK2A | 272/160 |
| 13 | CAMK2A | 326/168 |
| 14 | CAMK2A | 302/175 |
| 22 | CAMK2A | 202/53 |
| 25 | CAMK2A | 206/76 |
| 26 | CAMK2A | 371/221 |
| 27 | CAMK2A | 337/197 |
| 28 | CAMK2A | 287/127 |
| 10 | VGAT | 89/9 |
| 11 | VGAT | 57/12 |
| 16 | VGAT | 88/36 |
| 17 | VGAT | 103/21 |
| 18 | VGAT | 27/12 |
| 20 | VGAT | 67/14 |
| 23 | VGAT | 73/21 |
| 24 | VGAT | 89/19 |
| 29 | VGAT | 74/38 |
| 30 | VGAT | 124/21 |
| 31 | VGAT | 80/27 |
| 32 | VGAT | 85/29 |

**Supplemental Table 1.** Shown are the cell type, numbers of putative neurons and categorized place cells for each animal.

**Rotated Linear Track, non-rotation day (CAMK2A n=8, VGAT n=12)**

| **Mouse #** | **Cell Type** | **Cell count/Place cell count** |
| --- | --- | --- |
| 12 | CAMK2A | 260/42 |
| 13 | CAMK2A | 312/68 |
| 14 | CAMK2A | 304/77 |
| 22 | CAMK2A | 189/17 |
| 25 | CAMK2A | 201/56 |
| 26 | CAMK2A | 342/92 |
| 27 | CAMK2A | 325/113 |
| 28 | CAMK2A | 280/98 |
| 10 | VGAT | 89/11 |
| 11 | VGAT | 49/3 |
| 16 | VGAT | 90/20 |
| 17 | VGAT | 81/11 |
| 18 | VGAT | 28/3 |
| 20 | VGAT | 59/10 |
| 23 | VGAT | 75/5 |
| 24 | VGAT | 84/3 |
| 29 | VGAT | 71/10 |
| 30 | VGAT | 101/20 |
| 31 | VGAT | 82/3 |
| 32 | VGAT | 65/38 |

**Supplemental Table 2.** Shown are the cell type, numbers of putative neurons and categorized place cells for each animal.

**Rotated Linear Track, rotation day (CAMK2A n=8, VGAT n=11)**

| **Mouse #** | **Cell Type** | **Cell count/Place cell count** |
| --- | --- | --- |
| 12 | CAMK2A | 244/61 |
| 13 | CAMK2A | 248/84 |
| 14 | CAMK2A | 201/49 |
| 22 | CAMK2A | 124/15 |
| 25 | CAMK2A | 194/71 |
| 26 | CAMK2A | 224/124 |
| 27 | CAMK2A | 219/76 |
| 28 | CAMK2A | 194/81 |
| 11 | VGAT | 62/2 |
| 16 | VGAT | 57/9 |
| 17 | VGAT | 61/5 |
| 18 | VGAT | 45/4 |
| 20 | VGAT | 26/4 |
| 23 | VGAT | 52/8 |
| 24 | VGAT | 93/20 |
| 29 | VGAT | 88/3 |
| 30 | VGAT | 123/25 |
| 31 | VGAT | 85/4 |
| 32 | VGAT | 92/15 |

**Supplemental Table 3.** Shown are the cell type, numbers of putative neurons and categorized place cells for each animal.

**Short-Long Linear Track, M to M (CAMK2A n=8, VGAT n=12)**

| **Mouse #** | **Cell Type** | **Cell count/Place cell count** |
| --- | --- | --- |
| 12 | CAMK2A | 270/102 |
| 13 | CAMK2A | 321/106 |
| 14 | CAMK2A | 310/98 |
| 22 | CAMK2A | 195/8 |
| 25 | CAMK2A | 201/21 |
| 26 | CAMK2A | 360/118 |
| 27 | CAMK2A | 340/107 |
| 28 | CAMK2A | 284/98 |
| 10 | VGAT | 90/8 |
| 11 | VGAT | 52/8 |
| 16 | VGAT | 93/30 |
| 17 | VGAT | 95/10 |
| 18 | VGAT | 32/5 |
| 20 | VGAT | 62/6 |
| 23 | VGAT | 74/15 |
| 24 | VGAT | 85/10 |
| 29 | VGAT | 76/25 |
| 30 | VGAT | 122/17 |
| 31 | VGAT | 84/23 |
| 32 | VGAT | 81/25 |

**Supplemental Table 4.** Shown are the cell type, numbers of putative neurons and categorized place cells for each animal.

**Short-Long Linear Track, M to S (CAMK2A n=8, VGAT n=12)**

| **Mouse #** | **Cell Type** | **Cell count/Place cell count** |
| --- | --- | --- |
| 12 | CAMK2A | 184/45 |
| 13 | CAMK2A | 237/29 |
| 14 | CAMK2A | 196/117 |
| 22 | CAMK2A | 202/18 |
| 25 | CAMK2A | 266/64 |
| 26 | CAMK2A | 382/89 |
| 27 | CAMK2A | 291/110 |
| 28 | CAMK2A | 290/72 |
| 10 | VGAT | 80/21 |
| 11 | VGAT | 76/6 |
| 16 | VGAT | 73/37 |
| 17 | VGAT | 52/10 |
| 18 | VGAT | 29/2 |
| 20 | VGAT | 49/1 |
| 23 | VGAT | 70/26 |
| 24 | VGAT | 64/8 |
| 29 | VGAT | 68/23 |
| 30 | VGAT | 114/32 |
| 31 | VGAT | 64/27 |
| 32 | VGAT | 60/20 |

**Supplemental Table 5.** Shown are the cell type, numbers of putative neurons and categorized place cells for each animal.

**Short-Long Linear Track, M to L (CAMK2A n=8, VGAT n=12)**

| **Mouse #** | **Cell Type** | **Cell count/Place cell count** |
| --- | --- | --- |
| 12 | CAMK2A | 178/46 |
| 13 | CAMK2A | 149/135 |
| 14 | CAMK2A | 144/83 |
| 22 | CAMK2A | 167/52 |
| 25 | CAMK2A | 269/74 |
| 26 | CAMK2A | 349/154 |
| 27 | CAMK2A | 275/132 |
| 28 | CAMK2A | 291/87 |
| 10 | VGAT | 61/12 |
| 11 | VGAT | 49/2 |
| 16 | VGAT | 34/6 |
| 17 | VGAT | 20/1 |
| 18 | VGAT | 20/1 |
| 20 | VGAT | 46/7 |
| 23 | VGAT | 96/16 |
| 24 | VGAT | 76/21 |
| 29 | VGAT | 86/32 |
| 30 | VGAT | 107/28 |
| 31 | VGAT | 81/30 |
| 32 | VGAT | 78/13 |

**Supplemental Table 6.** Shown are the cell type, numbers of putative neurons and categorized place cells for each animal.

**Coregistered Sessions (CAMK2A n=7, VGAT n=8)**

| **Mouse #** | **Cell Type** | **Cell count (D1)** | **Cell count (D2)** | **Cell count (D3)** |
| --- | --- | --- | --- | --- |
| 12 | CAMK2A | 259 | 245 | 256 |
| 13 | CAMK2A | 242 | 322 | 346 |
| 14 | CAMK2A | 286 | 292 | 277 |
| 25 | CAMK2A | 241 | 261 | 260 |
| 26 | CAMK2A | 277 | 280 | 298 |
| 27 | CAMK2A | 314 | 324 | 306 |
| 28 | CAMK2A | 269 | 244 | 295 |
| 10 | VGAT | 58 | 60 | 64 |
| 11 | VGAT | 63 | 107 | 90 |
| 16 | VGAT | 69 | 59 | 55 |
| 17 | VGAT | 63 | 74 | 67 |
| 29 | VGAT | 86 | 67 | 68 |
| 30 | VGAT | 118 | 106 | 102 |
| 31 | VGAT | 87 | 77 | 70 |
| 32 | VGAT | 48 | 51 | 60 |

**Supplemental Table 7.** Shown are the cell type and number of putative neurons for each animal.
